# Different Modes of Anion Response Cause Circulatory Phase Transfer of a Coordination Cage with Controlled Directionality

**DOI:** 10.1002/anie.201906644

**Published:** 2019-07-30

**Authors:** Nozomi Mihara, Tanya K. Ronson, Jonathan R. Nitschke

**Affiliations:** ^1^ Department of Chemistry University of Cambridge Lensfield Road Cambridge CB2 1EW UK

**Keywords:** boranes, cage compounds, phase transfer, self-assembly, stimuli-responsiveness

## Abstract

Controlled directional transport of molecules is essential to complex natural systems, from cellular transport up to organismal circulatory systems. In contrast to these natural systems, synthetic systems that enable transport of molecules between several spatial locations on the macroscopic scale, when external stimuli are applied, remain to be explored. Now, the transfer of a supramolecular cage is reported with controlled directionality between three phases, based on a cage that responds reversibly in two distinct ways to different anions. Notably, circulatory phase transfer of the cage was demonstrated based on a system where the three layers of solvent are arranged within a circular track. The direction of circulation between solvent phases depended upon the order of addition of anions.

Transport of molecules between several spatial locations is essential to the functioning of complex natural systems. On a macroscopic scale, a circulatory system allows blood to transport nutrients throughout an organism. Scaled down to cellular transport, molecules are continuously transported between organelles, and in and out of the cell.^1^ Taking inspiration from these natural systems, it would be desirable to construct artificial systems where components are controllably transported between locations on a macroscopic scale, based on synthetic molecules that can interact with chemical signals, which induce different transport processes. In the present study, we demonstrate phase transfer of a stimuli‐responsive cage within a system consisting of three mutually immiscible solvent phases in which the direction of transfer is controlled by the order of application of distinct chemical signals. Notably, the cage showed circulatory phase transfer with controlled directionality when the three solvent phases were arranged in a circuit. This circulation could enable the development of new chemical purification systems involving the selective uptake and release of cargoes in specific spatial locations.^2^

Supramolecular cages^3^ and macrocycles^4^ are a versatile platform for the construction of stimuli‐responsive materials, since a wide variety of stimuli‐responsive subcomponents may be incorporated into them. The structure and electronic states of these assemblies can be altered by the application of stimuli that include electrons,^5^ light,^6^ pH,^7^ ions,^8^ and small molecules.^9^ This stimuli‐responsive behavior has been utilized to control functions that have included molecular recognition5c, 6a–6c, 8e and catalysis.8c, 8d A remaining important challenge in this field is the construction of supramolecular cages which respond to more than two stimuli in reversible and distinct ways, resulting in different outputs. Such cages could serve as building blocks for complex and functional supramolecular systems, where the output of one part serves as the input for another.

Herein we report the circulatory phase transfer of a newly synthesized Fe^II^_4_L_4_ tetrahedral cage containing tricoordinated boron atoms at the center of each face (Figure 1 a), in response to the addition of three different anions. Each such boron center shows selective and reversible binding of F^−^ to form a four‐coordinate fluoroborate,^10^ thus diminishing the total charge of the cage from 8+ to 4+. Since the cationic character of the cage also drives interaction with a variety of non‐coordinating anions, the phase preference of the cage can be controlled by the addition of different anions to induce transfer between specific pairs of phases. An anion grafted with perfluoroalkyl groups was found to solubilize the cage selectively in a fluorous phase, which made it possible to construct a triphasic system of immiscible solvent phases. Notably, the direction of the circulatory phase transfer of the cage can be controlled by changing the order of stimulus addition. Although phase transfer of molecules within conventional biphasic systems is well‐known,^11^ to the best of our knowledge this is the first example of a molecule that can be transferred within a triphasic solvent system in response to chemical stimuli.

**Figure 1:**
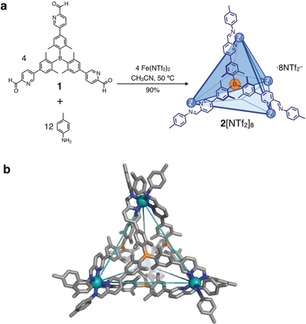
a) Cage **2** was prepared from tritopic aldehyde **1**, *p*‐toluidine, and iron(II) triflimide. b) The crystal structure of **2** (Fe, light blue; N, blue; B, orange; C, gray). Hydrogen atoms, counteranions, and disorder are omitted for clarity.

Borane‐containing tris(formylpyridine) **1** was prepared in three steps from 5‐bromo‐2‐iodo‐1,3‐xylene (Supporting Information, Sections S2.1 and S2.2). The reaction of **1** (4.0 equiv), *p*‐toluidine (12 equiv), and iron(II) bis(trifluoromethanesulfonyl)imide (iron(II) triflimide or Fe^II^(NTf_2_)_2_, 4.0 equiv) yielded Fe^II^_4_L_4_ assembly **2**[NTf_2_]_8_ (Figure 1 a), as confirmed by NMR spectroscopy and ESI‐MS (Figure 2 a; Supporting Information, Section S2.3). Vapor diffusion of benzene into an acetonitrile solution of **2**[NTf_2_]_8_ gave crystals suitable for structure determination by X‐ray diffraction. A representation of the X‐ray structure of **2** is shown in Figure 1 b.^12^ Four octahedral Fe^II^ centers are bridged by four ligands, each of which caps a face of the tetrahedron. All of the boron atoms have a planar sp^2^ configuration. The ligands on all faces of **2** have the same *C*_3_‐symmetric propeller‐like configuration in which the handedness of the propeller is the same as that of the Fe^II^ centers owing to the conformational rigidity of the cage framework.

**Figure 2:**
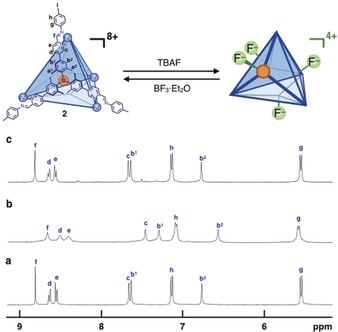
F^−^ binding of **2** in CD_3_CN. ^1^H NMR of a) **2**[NTf_2_]_8_, b) **2**[NTf_2_]_8_ after the addition of 4 equiv of TBAF to generate **2**⋅F_4_[NTf_2_]_4_, and c) the reaction mixture of **2**⋅F_4_[NTf_2_]_4_ with 4 equiv of BF_3_⋅Et_2_O.

To investigate the binding of F^−^ by **2**[NTf_2_]_8_, F^−^ was titrated into a solution of **2**[NTf_2_]_8_ (Figure 2 a,b, Figure S10). During the progressive addition of 4 equiv of tetrabutylammonium fluoride (TBAF) to **2**[NTf_2_]_8_ in CD_3_CN, the color of the solution changed from violet to green and new sets of ^1^H NMR peaks corresponding to adducts incorporating 1–4 equiv of F^−^ were observed. This observation indicates that the binding of F^−^ to the boron centers is slow on the ^1^H NMR time scale. A single set of ligand signals was obtained after the addition of 4 equiv of TBAF (Figure 2 b), consistent with the tetrafluoride adduct **2**⋅F_4_[NTf_2_]_4_ having a symmetric structure. In the ^19^F NMR spectra, a broad signal corresponding to F^−^ bound to boron was observed at −169 ppm during titration (Supporting Information, Figure S11), consistent with the formation of a B−F^−^ adduct.^13^ Since the methyl groups nearest the borane centers were observed as two distinct ^1^H NMR peaks, corresponding to inward‐ and outward‐facing methyl groups (Supporting Information, Figures S12 and S13), a ^1^H–^19^F HOESY experiment was undertaken to elucidate the geometry of the F^−^ group (Supporting Information, Figure S14). A NOE correlation was only observed between the externally‐oriented methyl resonance and the F^−^ signal, allowing us to conclude that all four F^−^ ions faced outward. ESI‐MS (Supporting Information, Figure S15) and UV/Vis titration (Supporting Information, Figure S16) also confirmed the binding of F^−^ to **2**.

Treatment with 4 equiv of BF_3_⋅Et_2_O was observed to immediately remove F^−^ from the boron atoms of **2**,^14^ resulting in a color change from green back to violet. Peaks corresponding to the original cage **2** were observed in the ^1^H NMR spectrum after the reaction (Figure 2 b,c; Supporting Information, Figure S17), as were ^19^F NMR signals corresponding to the co‐product BF_4_^−^ (Supporting Information, Figure S18). F^−^ binding can thus be used as a stimulus to reversibly change the structure and charge of **2**, which is the key feature allowing the circulatory phase transfer of **2**.

To construct a system composed of three mutually immiscible solvents, fluorous solvents were investigated as one of the solvent phases, since they are known to be immiscible with both water and many organic solvents.^15^ Although most nonfluorinated organic molecules are known not to dissolve in fluorous solvents, we found that a substituted tetraphenylborate bearing C_6_F_13_ chains (BAr_f6_^−^,^16^ Figure 3 a) imparted enough fluorous character^17^ (59 % fluorine content by weight) to render **2** preferentially soluble in fluorous solvents, even though the cage cation contains no fluorine. Counterion exchange of **2** from NTf_2_^−^ to BAr_f6_^−^ thus solubilized **2** in fluorous solvents such as perfluoromethylcyclohexane (PFMC, Supporting Information Section S6), perfluoro‐1,3‐dimethylcyclohexane, and perfluorohexane. Notably, this is the first example of a coordination cage that is soluble in fluorous solvents; we anticipate that the use of BAr_f6_^−^ could provide a general strategy to solubilize other cationic cages in fluorous phases.

**Figure 3:**
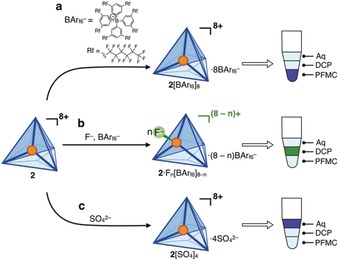
Stimuli‐responsive phase preferences of **2**. A triphasic solvent system composed of perfluoromethylcyclohexane (PFMC, densest), 2,2‐dichloropropane (DCP, middle), 25 % acetonitrile in water (Aq, least dense) was used to investigate the phase preference of **2** under the influence of different anionic stimuli. a) Highly fluorinated BAr_f6_^−^ brings **2** into fluorous PFMC. b) The presence of both F^−^ and BAr_f6_^−^ brings **2** into organic DCP. c) Hydrophilic SO_4_^2−^ solubilizes **2** in the aqueous phase.

Thus, a triphasic solvent system composed of PFMC, 2,2‐dichloropropane (DCP), and water containing 25 % acetonitrile was constructed, as shown in Figure 3. When both F^−^ and BAr_f6_^−^ were added, **2** was no longer soluble in the fluorous phase and became soluble in DCP (Figure 3 b). It was also found that SO_4_^2−^ could solubilize **2** in water containing 25 % acetonitrile, consistent with prior reports^18^ (Figure 3 c).

Stimuli could bring cage **2** from any of the phases of Figure 3 into any other phase. Starting with **2**[BAr_f6_]_8_ in fluorous PFMC, addition of TBAF (4.0 equiv) to the triphasic system resulted in **2** transferring to organic DCP (Figure 4 a). During this process, the color of **2** changed from violet to green, consistent with the formation of the F^−^ adduct of **2** as described above. The selective binding of F^−^ to **2** results in a reduced degree of ion pairing with fluorous BAr_f6_^−^. We infer that the resulting fluoride adduct no longer has sufficient fluorous character to be soluble in PFMC. The addition of tetrabutylammonium sulfate (TBA_2_SO_4_, 3.0 equiv), and MgSO_4_ (2.0 equiv) resulted in transfer of **2** to the aqueous phase, with the color changing back to violet (Figure 4 b). F^−^ was removed from the system by precipitation as MgF_2_ in this step.^19^ This phase‐transfer cycle could be repeated a second time by the sequential addition of NaBAr_f6_, then TBAF, and finally TBA_2_SO_4_, and MgSO_4_ (Figure 4 c–e).

**Figure 4:**
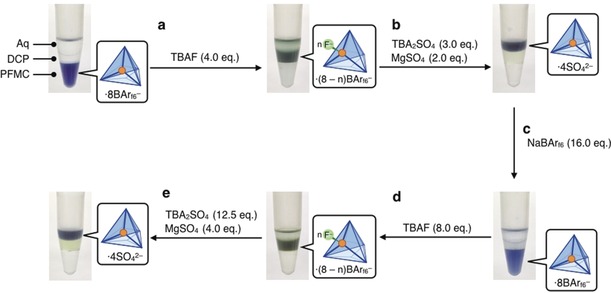
Transport experiment of **2** within the triphasic solvent system. a) Addition of TBAF to **2**[BAr_f6_]_8_ in PFMC resulted in cage transfer to DCP. b) Subsequent addition of TBA_2_SO_4_ and MgSO_4_ led to the transport of **2** to the aqueous phase. c)–e) Progressive addition of NaBAr_f6_, then TBAF, and finally TBA_2_SO_4_, and MgSO_4_ led to a second cycle of transport.

Conversely, when TBA_2_SO_4_ (4.0 equiv) was added to the initial state of the triphasic system, cage **2** transferred to the aqueous phase (Supporting Information, Figure S25 a‐(ii)), then to DCP following the addition of TBAF (8.0 equiv) and NaBAr_f6_ (8.9 equiv; Supporting Information, Figure S25 a‐(iii)). This cycle could also be repeated following the successive additions of the different salts (Supporting Information, Figure S25 a‐(iv)–(vi)). These results demonstrate that cage **2** underwent directional and reversible transport within the triphasic system depending on the order of addition of anions. Further details of the transfer process including the efficiency are presented in the Supporting Information, Sections S7.1–S7.3.

Based upon these results, we designed a platform for circulatory phase transfer by arranging the three solvent layers within a circular glass tube with three injection points, as shown in Figure 5. The transport experiment was performed in a similar manner to the experiments in microtubes (Supporting Information, Section S7.4). To the initial state, with **2**[BAr_f6_]_8_ in fluorous PFMC, TBAF (4.0 equiv) was added (Figure 5 a). Gentle agitation resulted in the transportation of **2** from PFMC to DCP. Subsequent addition of TBA_2_SO_4_ (3.0 equiv) and MgSO_4_ (2.0 equiv) resulted in the transfer of **2** to the aqueous phase (Figure 5 b). Cage **2** thus moved in a clockwise direction through the circuit. On the other hand, when TBA_2_SO_4_ (4.0 equiv) was first added, followed by TBAF (8.0 equiv) with NaBAr_f6_ (8.9 equiv), **2** circulated in an anticlockwise direction (first to the aqueous phase and then to DCP, (Figure 5 c,d)). The order of applied stimuli thus enabled control over the direction of circulatory phase transfer.

**Figure 5:**
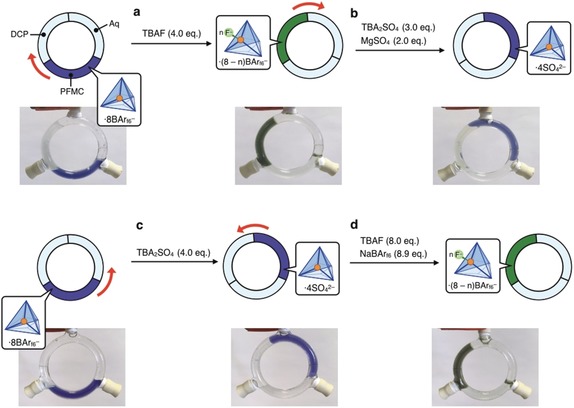
Phase transfer of **2** within a circuit. a),b) When TBAF was first added and TBA_2_SO_4_ and MgSO_4_ were added subsequently, **2** was first transported to DCP and then to the aqueous phase, in a clockwise direction. c),d) When TBA_2_SO_4_ was added first and TBAF and NaBAr_f6_ were added subsequently, **2** moved to the aqueous phase first and then DCP, showing anticlockwise transfer.

This study thus establishes the circulatory phase transfer of stimuli‐responsive coordination cage **2**. Directional control over circulatory phase transfer depends upon the responsiveness of **2** to three stimuli: fluoride, the highly fluorinated anion BAr_f6_^−^, and sulfate. The selective fluoride binding to the borane centers is the key feature enabling the phase preference of **2** to be switched. Notably, we present the first example of a coordination cage solubilized in fluorous solvents by utilizing BAr_f6_^−^, which could form the basis of a general strategy for other polycationic cages, which may enable the exploration of new guest recognition phenomena in fluorous solvents. Larger cages than **2**, which would show the ability to bind a range of guest molecules, could be constructed by incorporating the central borane motif of **1** into larger ligand panels. Such larger cages could be built into more sophisticated circulatory guest transport systems. The ability to transit reversibly between three different phases could allow **2** and its analogues to be directed along complex pathways within a fluid network of linked rings, or circulatory phase transfer could be used to pick up a guest in one phase, then move it to another where the cage might open to release its cargo.^2^ Such circulatory guest transport could be useful as a new mode of chemical purification.

## Conflict of interest

The authors declare no conflict of interest.

## Supporting information

As a service to our authors and readers, this journal provides supporting information supplied by the authors. Such materials are peer reviewed and may be re‐organized for online delivery, but are not copy‐edited or typeset. Technical support issues arising from supporting information (other than missing files) should be addressed to the authors.

Supplementary

## References

[1a] J. E. Rothman , F. T. Wieland , Science 1996, 272, 227–234; 8602507 10.1126/science.272.5259.227

[1b] P. Novick , M. Zerial , Curr. Opin. Cell Biol. 1997, 9, 496–504; 9261061 10.1016/s0955-0674(97)80025-7

[1c] K. Sato , A. Nakano , FEBS Lett. 2007, 581, 2076–2082. 17316621 10.1016/j.febslet.2007.01.091

[2] D. Zhang , T. K. Ronson , J. Mosquera , A. Martinez , J. R. Nitschke , Angew. Chem. Int. Ed. 2018, 57, 3717–3721; 10.1002/anie.201800459 PMC6001518 29393989

[3a] M. Yoshizawa , M. Tamura , M. Fujita , Science 2006, 312, 251–254; 16614218 10.1126/science.1124985

[3b] L. Avram , Y. Cohen , J. Rebek, Jr. , Chem. Commun. 2011, 47, 5368–5375; 10.1039/c1cc10150a 21431233

[3c] T. Nakamura , H. Ube , M. Shiro , M. Shionoya , Angew. Chem. Int. Ed. 2013, 52, 720–723; 10.1002/anie.201208040 23161786

[3d] T. R. Cook , P. J. Stang , Chem. Rev. 2015, 115, 7001–7045; 25813093 10.1021/cr5005666

[3e] W. Cullen , M. C. Misuraca , C. A. Hunter , N. H. Williams , M. D. Ward , Nat. Chem. 2016, 8, 231–236; 26892554 10.1038/nchem.2452

[3f] T. H. G. Schick , J. C. Lauer , F. Rominger , M. Mastalerz , Angew. Chem. Int. Ed. 2019, 58, 1768–1773; 10.1002/anie.201814243 PMC6470955 30557460

[4a] P. D. Frischmann , B. J. Sahli , S. Guieu , B. O. Patrick , M. J. MacLachlan , Chem. Eur. J. 2012, 18, 13712–13721; 22997016 10.1002/chem.201201536

[4b] Z. J. Kinney , C. S. Hartley , J. Am. Chem. Soc. 2017, 139, 4821–4827; 28304166 10.1021/jacs.7b00149

[4c] G. Yu , X. Zhao , J. Zhou , Z. Mao , X. Huang , Z. Wang , B. Hua , Y. Liu , F. Zhang , Z. He , O. Jacobson , C. Gao , W. Wang , C. Yu , X. Zhu , F. Huang , X. Chen , J. Am. Chem. Soc. 2018, 140, 8005–8019; 29874067 10.1021/jacs.8b04400

[4d] K. Jie , Y. Zhou , E. Li , R. Zhao , F. Huang , Angew. Chem. Int. Ed. 2018, 57, 12845–12849; 10.1002/anie.201808998 30088688

[5a] A. C. Fahrenbach , C. J. Bruns , H. Li , A. Trabolsi , A. Coskun , J. F. Stoddart , Acc. Chem. Res. 2014, 47, 482–493; 24341283 10.1021/ar400161z

[5b] M. J. Burke , G. S. Nichol , P. J. Lusby , J. Am. Chem. Soc. 2016, 138, 9308–9315; 27351912 10.1021/jacs.6b05364

[5c] G. Szalóki , V. Croué , V. Carré , F. Aubriet , O. Alévêque , E. Levillain , M. Allain , J. Aragó , E. Ortí , S. Goeb , M. Sallé , Angew. Chem. Int. Ed. 2017, 56, 16272–16276; 10.1002/anie.201709483 29083516

[5d] S. Wang , Z. Xu , T. Wang , T. Xiao , X.-Y. Hu , Y.-Z. Shen , L. Wang , Nat. Commun. 2018, 9, 1737. 29712901 10.1038/s41467-018-03827-3 PMC5928112

[6a] T. Murase , S. Sato , M. Fujita , Angew. Chem. Int. Ed. 2007, 46, 5133–5136; 10.1002/anie.200700793 17535006

[6b] M. Han , R. Michel , B. He , Y.-S. Chen , D. Stalke , M. John , G. H. Clever , Angew. Chem. Int. Ed. 2013, 52, 1319–1323; 10.1002/anie.201207373 23208865

[6c] N. Kishi , M. Akita , M. Kamiya , S. Hayashi , H.-F. Hsu , M. Yoshizawa , J. Am. Chem. Soc. 2013, 135, 12976–12979; 23957216 10.1021/ja406893y

[6d] X.-F. Jiang , F. K.-W. Hau , Q.-F. Sun , S.-Y. Yu , V. W.-W. Yam , J. Am. Chem. Soc. 2014, 136, 10921–10929; 25062467 10.1021/ja502295c

[6e] S. Chen , L.-J. Chen , H.-B. Yang , H. Tian , W. Zhu , J. Am. Chem. Soc. 2012, 134, 13596–13599; 22881042 10.1021/ja306748k

[6f] X. Yan , J.-F. Xu , T. R. Cook , F. Huang , Q.-Z. Yang , C.-H. Tung , P. J. Stang , Proc. Natl. Acad. Sci. USA 2014, 111, 8717–8722; 24889610 10.1073/pnas.1408620111 PMC4066485

[6g] J. Park , L.-B. Sun , Y.-P. Chen , Z. Perry , H.-C. Zhou , Angew. Chem. Int. Ed. 2014, 53, 5842–5846; 10.1002/anie.201310211 24803325

[6h] D. Preston , J. J. Sutton , K. C. Gordon , J. D. Crowley , Angew. Chem. Int. Ed. 2018, 57, 8659–8663; 10.1002/anie.201804745 29774643

[7a] J. E. M. Lewis , E. L. Gavey , S. A. Cameron , J. D. Crowley , Chem. Sci. 2012, 3, 778–784;

[7b] M. Ni , N. Zhang , W. Xia , X. Wu , C. Yao , X. Liu , X.-Y. Hu , C. Lin , L. Wang , J. Am. Chem. Soc. 2016, 138, 6643–6649; 27159331 10.1021/jacs.6b03296

[7c] M. Cheng , J. Zhang , X. Ren , S. Guo , T. Xiao , X.-Y. Hu , J. Jiang , L. Wang , Chem. Commun. 2017, 53, 11838–11841; 10.1039/c7cc07469g 29039857

[7d] L.-Y. Sun , N. Sinha , T. Yan , Y.-S. Wang , T. T. Y. Tan , L. Yu , Y.-F. Han , F. E. Hahn , Angew. Chem. Int. Ed. 2018, 57, 5161–5165; 10.1002/anie.201713240 29394472

[8a] Z. Qi , C. Wu , P. Malo de Molina , H. Sun , A. Schulz , C. Griesinger , M. Gradzielski , R. Haag , M. B. Ansorge-Schumacher , C. A. Schalley , Chem. Eur. J. 2013, 19, 10150–10159; 23843281 10.1002/chem.201300193

[8b] F. Durola , V. Heitz , F. Reviriego , C. Roche , J.-P. Sauvage , A. Sour , Y. Trolez , Acc. Chem. Res. 2014, 47, 633–645; 24428574 10.1021/ar4002153

[8c] A. M. Lifschitz , M. S. Rosen , C. M. McGuirk , C. A. Mirkin , J. Am. Chem. Soc. 2015, 137, 7252–7261; 26035450 10.1021/jacs.5b01054

[8d] M. Galli , E. M. Lewis , S. M. Goldup , Angew. Chem. Int. Ed. 2015, 54, 13545–13549; 10.1002/anie.201505464 PMC4678423 26387887

[8e] Y. Sakata , C. Murata , S. Akine , Nat. Commun. 2017, 8, 16005; 28699635 10.1038/ncomms16005 PMC5510176

[8f] X.-Q. Wang , W. Wang , W.-J. Li , L.-J. Chen , R. Yao , G.-Q. Yin , Y.-X. Wang , Y. Zhang , J. Huang , H. Tan , Y. Yu , X. Li , L. Xu , H.-B. Yang , Nat. Commun. 2018, 9, 3190; 30093667 10.1038/s41467-018-05670-y PMC6085385

[8g] R. Djemili , L. Kocher , S. Durot , A. Peuroren , K. Rissanen , V. Heitz , Chem. Eur. J. 2019, 25, 1481–1487. 30536482 10.1002/chem.201805498

[9a] H. L. Ozores , M. Amorín , J. R. Granja , J. Am. Chem. Soc. 2017, 139, 776–784; 27996247 10.1021/jacs.6b10456

[9b] M. Zhang , M. L. Saha , M. Wang , Z. Zhou , B. Song , C. Lu , X. Yan , X. Li , F. Huang , S. Yin , P. J. Stang , J. Am. Chem. Soc. 2017, 139, 5067–5074. 28332834 10.1021/jacs.6b12536

[10a] S. Yamaguchi , S. Akiyama , K. Tamao , J. Am. Chem. Soc. 2001, 123, 11372–11375; 11707112 10.1021/ja015957w

[10b] S.-T. Lam , N. Zhu , V. W.-W. Yam , Inorg. Chem. 2009, 48, 9664–9670; 19746985 10.1021/ic900803a

[10c] C. R. Wade , A. E. J. Broomsgrove , S. Aldridge , F. P. Gabbaï , Chem. Rev. 2010, 110, 3958–3984. 20540560 10.1021/cr900401a

[11a] T. J. Ryan , G. Lecollinet , T. Velasco , A. P. Davis , Proc. Natl. Acad. Sci. USA 2002, 99, 4863–4866; 11929965 10.1073/pnas.062013499 PMC122684

[11b] Q. Lin , H. J. Yun , W. Liu , H.-J. Song , N. S. Makarov , O. Isaienko , T. Nakotte , G. Chen , H. Luo , V. I. Klimov , J. M. Pietryga , J. Am. Chem. Soc. 2017, 139, 6644–6653; 28431206 10.1021/jacs.7b01327

[11c] S.-Y. Zhang , Z. Kochovski , H.-C. Lee , Y. Lu , H. Zhang , J. Zhang , J.-K. Sun , J. Yuan , Chem. Sci. 2019, 10, 1450–1456. 30809362 10.1039/c8sc04375b PMC6354838

[12] CCDC 1881666 contains the supplementary crystallographic data for this paper. These data can be obtained free of charge from The Cambridge Crystallographic Data Centre.

[13] Y. Hu , M. Kye , J. Y. Jung , Y.-b. Lim , J. Yoon , Sens. Actuators B 2018, 255, 2621–2627.

[14] Z. Zhou , A. Wakamiya , T. Kushida , S. Yamaguchi , J. Am. Chem. Soc. 2012, 134, 4529–4532. 22369126 10.1021/ja211944q

[15] P. Babiak , A. Němcová , L. Rulíšek , P. Beier , J. Fluorine Chem. 2008, 129, 397–401.

[16] J. van den Broeke , B.-J. Deelman , G. van Koten , Tetrahedron Lett. 2001, 42, 8085–8087.

[17] W. Zhang , Chem. Rev. 2004, 104, 2531–2556. 15137799 10.1021/cr030600r PMC1618880

[18a] E. G. Percástegui , J. Mosquera , T. K. Ronson , A. J. Plajer , M. Kieffer , J. R. Nitschke , Chem. Sci. 2019, 10, 2006–2018; 30881630 10.1039/c8sc05085f PMC6385555

[18b] A. B. Grommet , J. B. Hoffman , E. G. Percástegui , J. Mosquera , D. J. Howe , J. L. Bolliger , J. R. Nitschke , J. Am. Chem. Soc. 2018, 140, 14770–14776. 30371068 10.1021/jacs.8b07900

[19] BF_3_⋅Et_2_O could not be used to remove F^−^ for this triphasic system because of the reactivity to water. Insolubility of MgF_2_ was confirmed by ^19^F NMR of a suspension of MgF_2_ in D_2_O containing 25 % CD_3_CN, where no signal corresponding to F^−^ was observed.

