# Supplementary material for: Different Modes of Anion Response Cause Circulatory Phase Transfer of a Coordination Cage with Controlled Directionality
Source: Angew Chem Int Ed Engl. 2019 Jul 30;58(36):12497–501. doi: 10.1002/anie.201906644 (PMC6771743; doi:10.1002/anie.201906644)
Supplement: Supplementary file 1 — Supplementary [file ANIE-58-12497-s001.pdf]

Angewandte Chemie
Eine Zeitschrift der Gesellschaft Deutscher Chemiker

Supporting Information

Different Modes of Anion Response Cause Circulatory Phase Transfer of a Coordination Cage with Controlled Directionality

*Nozomi Mihara, Tanya K. Ronson, and Jonathan R. Nitschke**

anie_201906644_sm_miscellaneous_information.pdf

Table of contents

S1 General methods S3

S2 Synthesis and characterization S4
S2.1 Synthesis of **5** (Figure S1) S4
S2.2 Synthesis of **1** (Figure S2, S3) S5
S2.3 Synthesis of $Fe^{II}_4L_4$ cage **2**$[NTf_2]_8$ (Figure S4 – S9) S7

S3 X-ray crystallography of 2$[NTf_2]_8$ S11

S4 F^- binding of cage 2$[NTf_2]_8$ S13
S4.1 NMR titration of **2**$[NTf_2]_8$ with F^- (Figure S10 – S15) S13
S4.2 UV-Vis titration of **2**$[NTf_2]_8$ with F^- (Figure S16) S17

S5 Removal of F^- from 2·$F_4[NTf_2]_4$ (Figure S17, S18) S18

S6 Counter anion exchange of 2$[NTf_2]_8$ to 2$[BAr_{f6}]_8$ (Figure S19 – S21) S20

S7 Transport experiment of 2 within triphasic solvent system S22
S7.1 Calibration curves for transport experiment (Figure S22) S22
S7.2 UV-Vis titration of **2** with F^- for transport experiment (Figure S23) S23
S7.3 Transport experiment of **2** in a microtube (Figure S24 – S27) S24
S7.3.1 Experimental procedure S24
S7.3.2 Experimental results and discussion about transport process S25
S7.4 Transport experiment of **2** in a circular glass tube S31

S8 References S32

S1 General methods

NMR spectra were recorded using a 400 MHz Advance III HD Smart Probe, DPX S5 500 MHz BB ATM. Chemical shifts for ^{1}H, ^{13}C and ^{19}F NMR are reported in ppm on the δ scale; ^{1}H and ^{13}C were referenced to the residual solvent peak and ^{19}F was referenced to an internal standard of C_6F_6 in CD_3CN at −164.9 ppm. Coupling constants (J) are reported in hertz (Hz). The following abbreviations are used to describe signal multiplicity for ^{1}H, ^{13}C and ^{19}F NMR spectra: s: singlet, d: doublet, t: triplet, dd: doublet of doublets; dt: doublet of triplets; m: multiplet, br: broad.

UV-Visible absorption spectroscopy was performed at 295 K using a Perkin Elmer Lambda 750 UV-Vis-NIR spectrophotometer operating in double beam mode. Samples were analysed using quartz cuvettes with optical path lengths of 1mm.

High resolution mass spectrometry for **1** was performed on a Waters LCT Premier Mass Spectrometer featuring a Z-spray source with electrospray ionisation and modular LockSpray interface. High resolution mass spectrometric experiment for **2**$[NTf_2]_8$ was obtained on a Thermofisher LTQ Orbitrap XL hybrid ion trap mass spectrometer with electrospray ionisation. Low resolution electrospray ionisation (ESI) mass spectra for **2**$\cdot F_4[NTf_2]_4$ and the transport experiments were obtained on a Micromass Quattro LC mass spectrometer infused from a Harvard syringe pump at a rate of 10 μL min^{-1}.

Compound **3**,[1] iron(II) bis(trifluoromethane)sulfonimide,[2] and $NaBAr_{f6}$[3] were synthesized according to reported procedures. Compound **4** was synthesized by a similar method as reported before[4].

S2 Synthesis and characterization

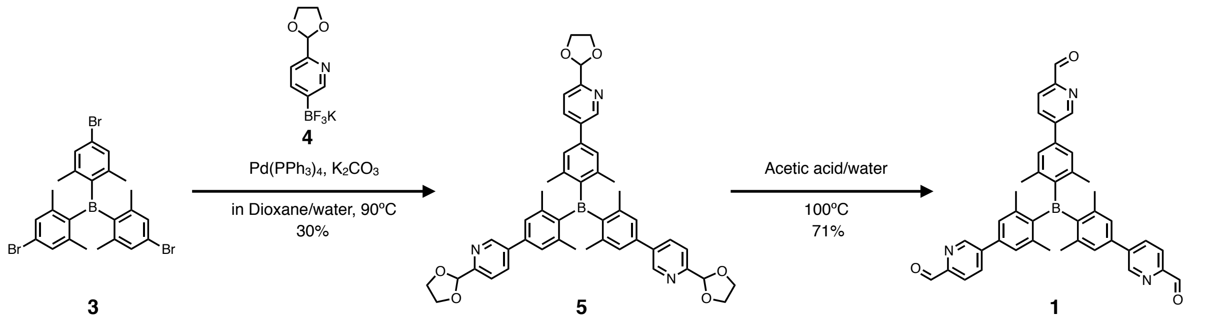

Scheme S1 Synthesis of ligand 1.

S2.1 Synthesis of 5

A mixture of **3** (400 mg, 0.710 mmol), **4** (603 mg, 2.34 mmol), $Pd(PPh_3)_4$ (240 mg, 0.280 mmol) and K_2CO_3 (772 mg, 5.59 mmol) in 1,4-dioxane (18 mL) and water (4.5 mL) was degassed and stirred under a N_2 atmosphere at 90 ºC for 4 hrs. The resulting mixture was diluted by CH_2Cl_2 (100 mL) and washed with water (100 mL × 2) and brine (50 mL). The crude product was purified by column chromatography on silica gel (eluent: AcOEt). The fraction containing **5** was evaporated and washed with AcOEt to obtain the title compound **5** as a colorless solid (163 mg, 30%). ^{1}H NMR (δ (ppm), 400 MHz, $CDCl_3$): 8.88 (d, J = 1.9 Hz, 3H), 7.96 (dd, J = 8.1 Hz, 2.2 Hz, 3H), 7.60 (d, J = 8.0 Hz, 3H), 7.20 (s, 6H), 5.92 (s, 3H), 4.23-4.09 (m, 12H), 2.16 (s, 18H).

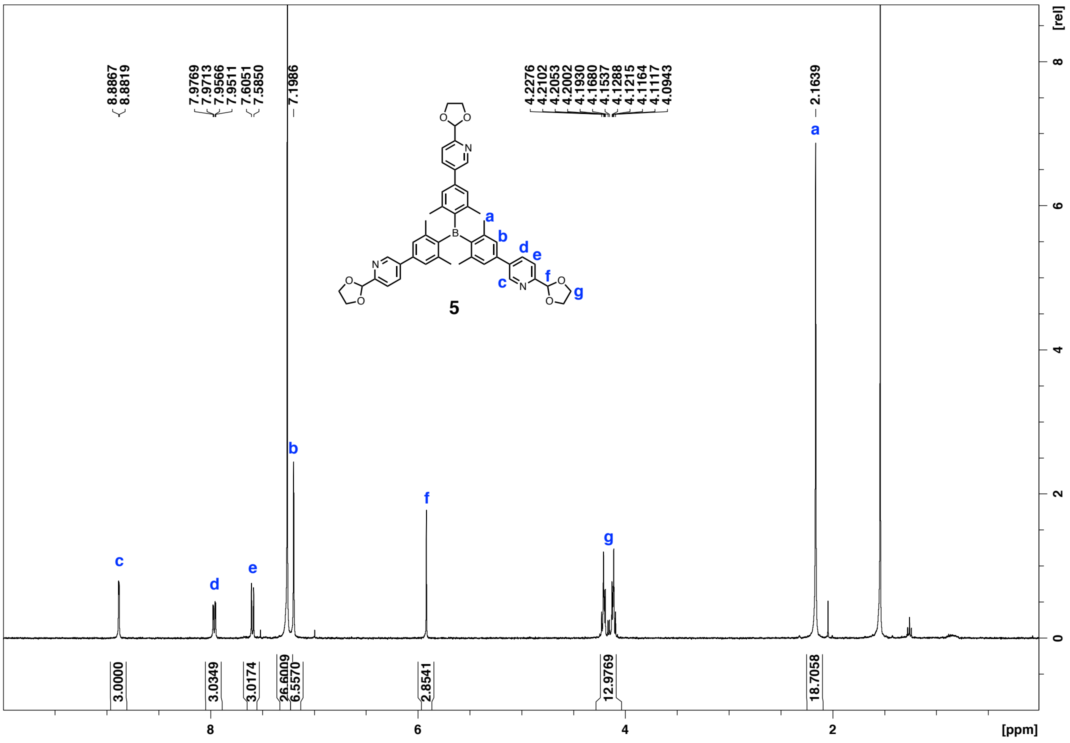

Figure S1 ^{1}H NMR (400 MHz) of **5** in $CDCl_3$ at room temperature.

S2.2 Synthesis of 1

A mixture of **5** (163 mg, 0.211 mmol) in acetic acid (3.0 mL) and water (0.6 mL) was stirred at 100 °C for 2 hr. The resulting solution was neutralized by addition of sat. $NaHCO_3$ aq. and extracted with CH_2Cl_2 (100 mL × 2). The combined organic layers were dried by $MgSO_4$ and evaporated. The crude product was purified by column chromatography on silica gel (eluent: CH_2Cl_2 : AcOEt = 3 : 1) to obtain the title compound **1** as a pale yellow solid (96 mg, 71%). ^{1}H NMR (*δ* (ppm), 400 MHz, $CDCl_3$): 10.1 (s, 3H), 9.06 (d, *J* = 1.7 Hz, 3H), 8.11 (dd, *J* = 8.1 Hz, 1.9 Hz, 3H), 8.04 (d, *J* = 8.1 Hz, 3H), 7.29 (s, 6H), 2.20 (s, 18H). ^{13}C NMR (δ (ppm), 100 MHz, $CDCl_3$): 193.2, 151.8, 148.7, 147.2, 141.8, 140.3, 137.9, 135.1, 127.0, 122.0, 23.4. HR-MS (ESI-Q-TOF, positive) *m/z* calcd. for $(\mathbf{1}+H^+)^+$ 642.2922, found 642.2924.

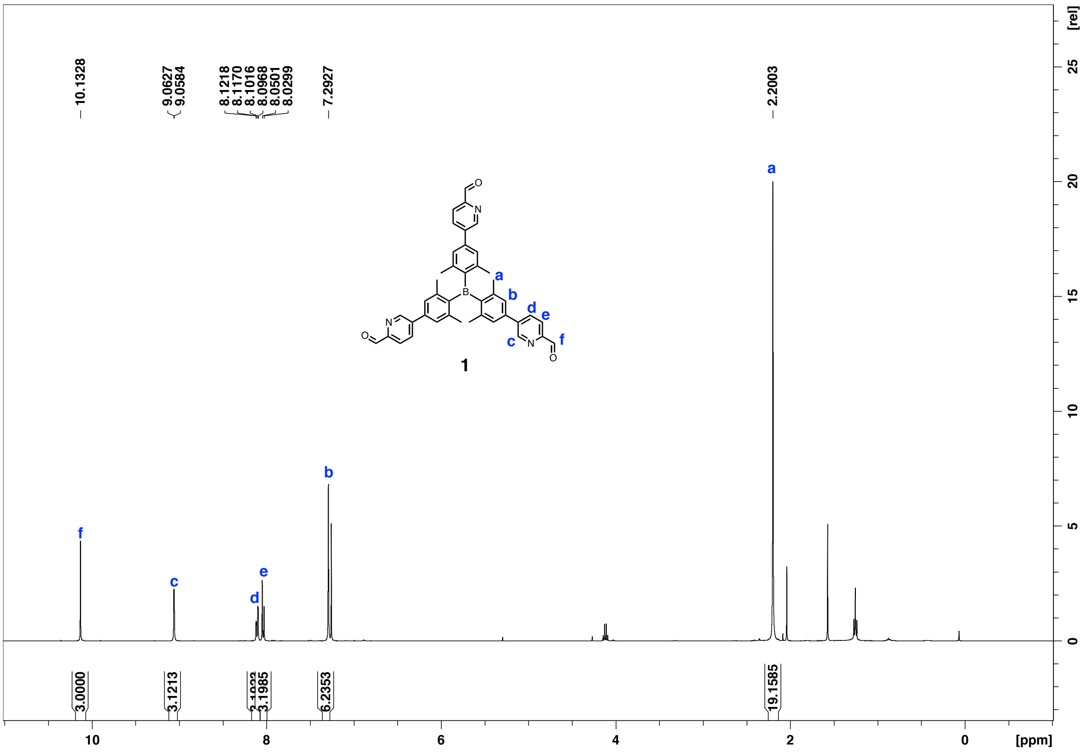

Figure S2 ^{1}H NMR (400 MHz) of **1** in $CDCl_3$ at room temperature.

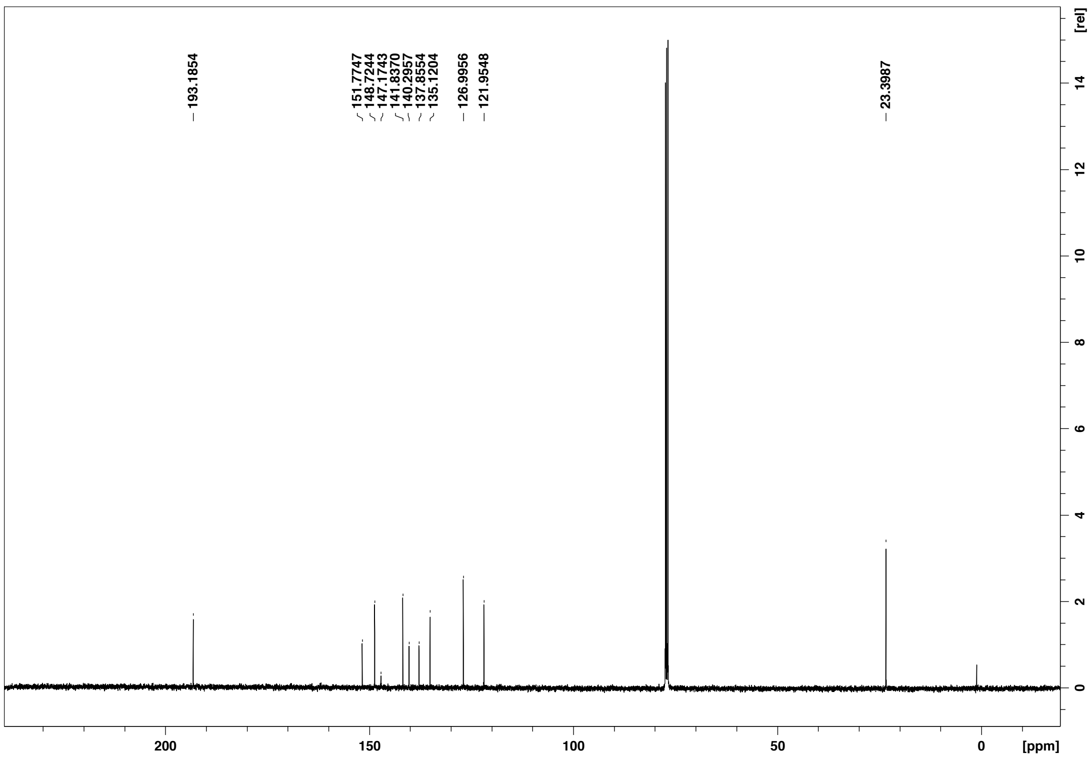

Figure S3 ^{13}C NMR (100 MHz) of **1** in $CDCl_3$ at room temperature.

S2.3 Characterization of $Fe^{II}_4L_4$ cage 2[NTf$_2$]$_8$

Trialdehyde **1** (80.0 mg, 125 μmol), p-toluidine (42.8 mg, 399 μmol), and $Fe(NTf_2)_2$·4.5 H_2O (85.6 mg, 123 μmol) in dry acetonitrile (80 mL) were degassed and heated at 50 °C under a N_2 atmosphere for 24 hrs. The title compound **2**[NTf$_2$]$_8$ was precipitated by addition of diethyl ether to the reaction mixture at room temperature (172 mg, 90%). ^{1}H NMR (*δ* (ppm), 400 MHz, CD_3CN): 8.77 (s, 12H), 8.59 (d, *J* = 6.4 Hz, 12H), 8.51 (d, *J* = 6.5 Hz, 12H), 7.62 (s, 12H), 7.59 (s, 12H), 7.09 (d, *J* = 6.4 Hz, 24H), 6.72 (s, 12H), 5.51 (d, *J* = 6.3 Hz, 24H), 2.38 (s, 36H), 2.11 (s, 36H), 1.46 (s, 36H). ^{13}C NMR (δ (ppm), 100 MHz, CD_3CN): 174.8, 158.8, 152.3, 149.0, 148.2, 144.4, 141.3, 141.2, 140.1, 138.6, 137.1, 132.1, 130.8, 128.1, 126.7, 122.4, 120.9, 23.3, 21.0. HR-MS (ESI-Q-TOF, positive) *m/z* calcd. for (**2**[NTf$_2$])$^{7+}$ 591.37, found 591.37; calcd. for (**2**[NTf$_2$]$_2$)$^{6+}$ 736.58, found 736.58; calcd. for (**2**[NTf$_2$]$_3$)$^{5+}$ 939.88, found 939.88; calcd. for (**2**[NTf$_2$]$_4$)$^{4+}$ 1244.83, found 1244.83; calcd. for (**2**[NTf$_2$]$_5$)$^{3+}$ 1753.08, found 1753.08. Anal. Calcd for $C_{268}H_{228}B_4F_{48}Fe_4N_{32}O_{32}S_{16}$ (**2**[NTf$_2$]$_8$): C, 52.76; H, 3.77; N, 7.35. Found: C, 52.60; H, 3.88; N, 7.17 (0.18% error).

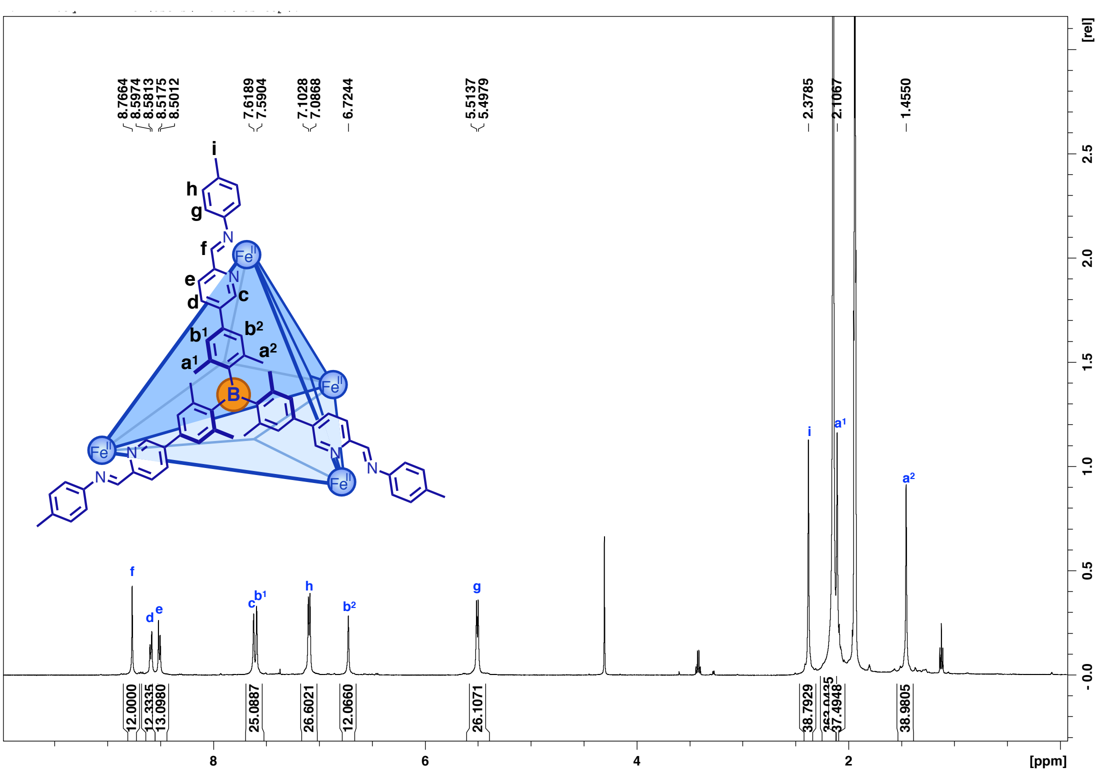

Figure S4 ^{1}H NMR (400 MHz) of **2**[NTf$_2$]$_8$ in CD_3CN at room temperature.

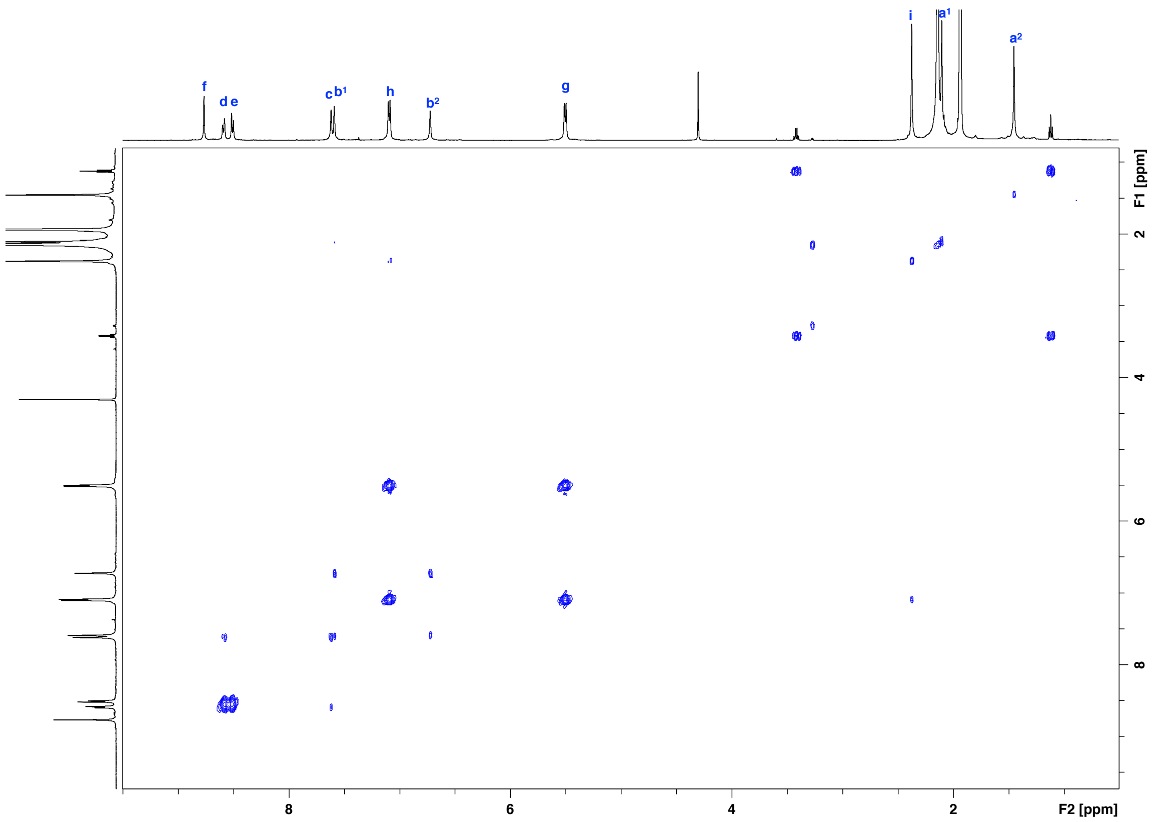

Figure S5 ^{1}H–^{1}H COSY (400 MHz) of **2**$[NTf_2]_8$ in CD_3CN at room temperature.

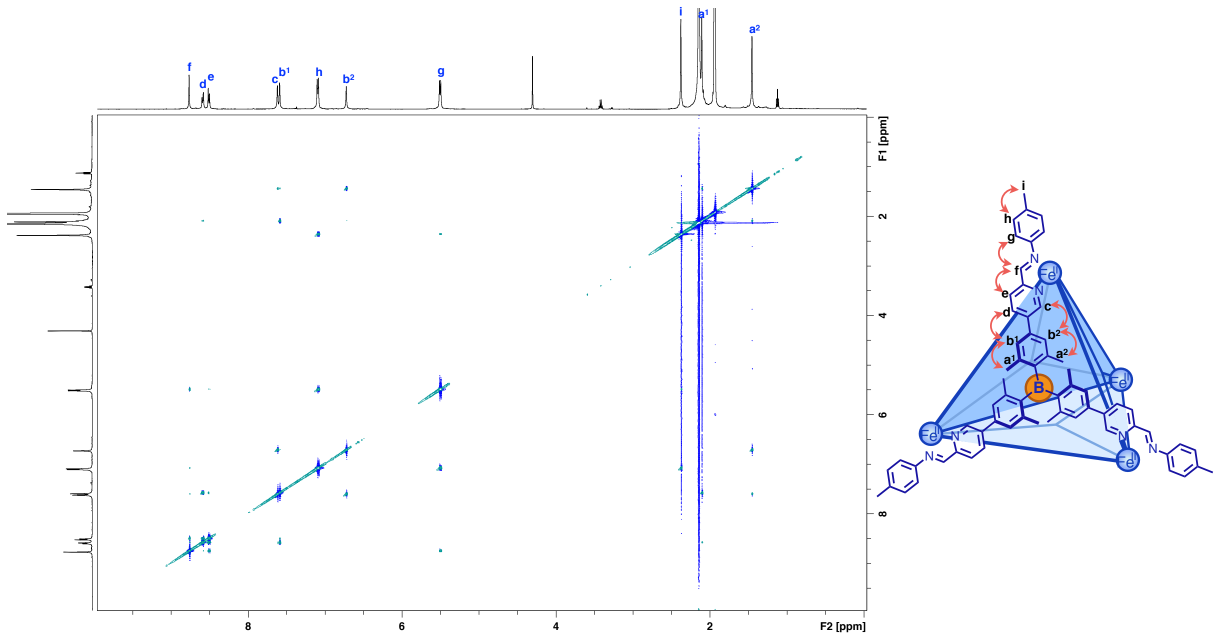

Figure S6 ^{1}H–^{1}H NOESY (400 MHz) of **2**$[NTf_2]_8$ in CD_3CN at room temperature.

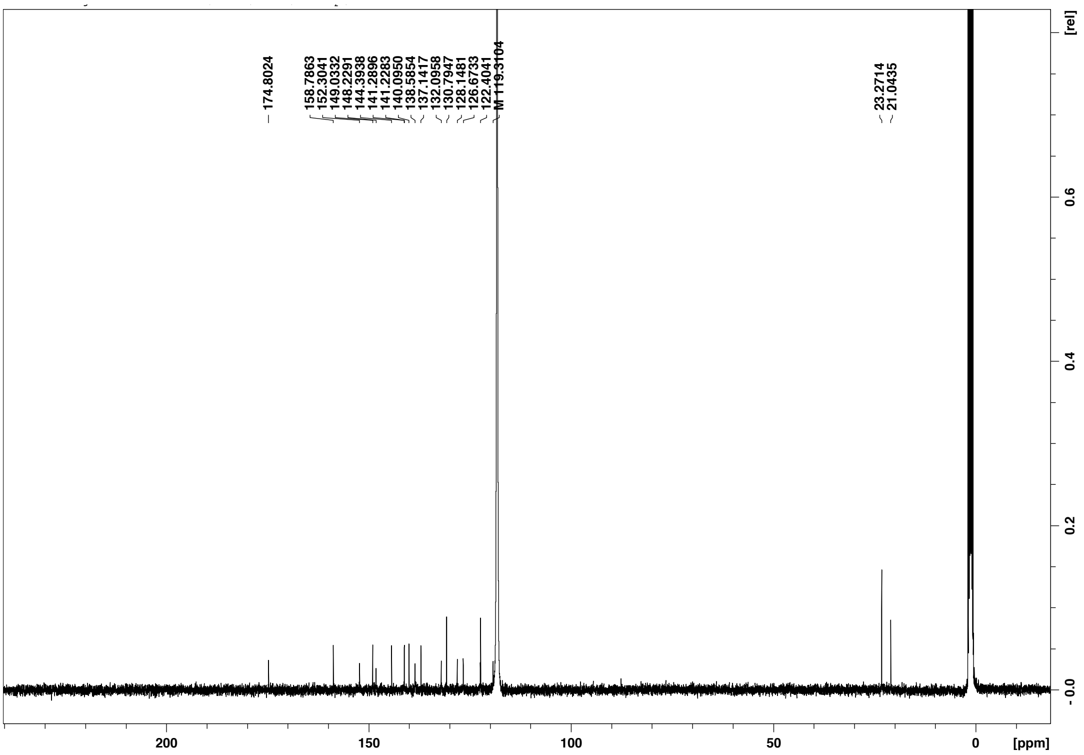

Figure S7 ^{13}C NMR (100 MHz) of **2**$[NTf_2]_8$ in CD_3CN at room temperature. We infer that two of the three CH_3 signals (**a**^{1}, **a**^{2}, **i**) are overlapping as the signal at 23.3 ppm. The quartet expected for NTf_2^- could not be clearly resolved due to the low signal intensity.

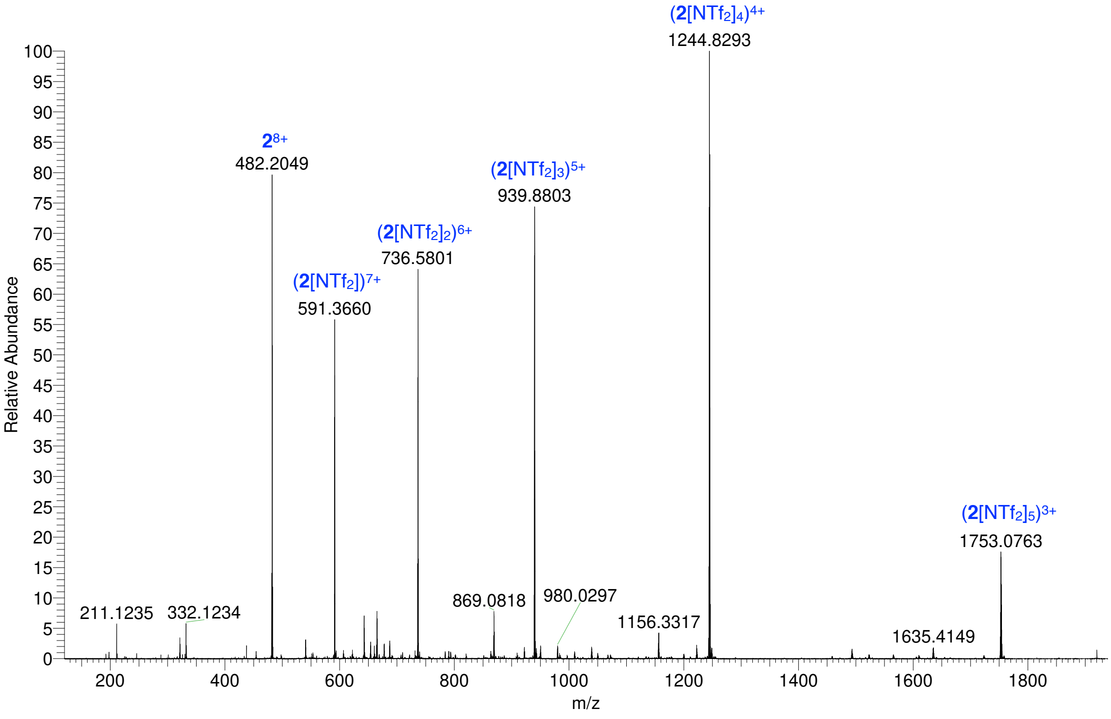

Figure S8 High resolution ESI-TOF-MS of **2**[NTf_2]$_8$.

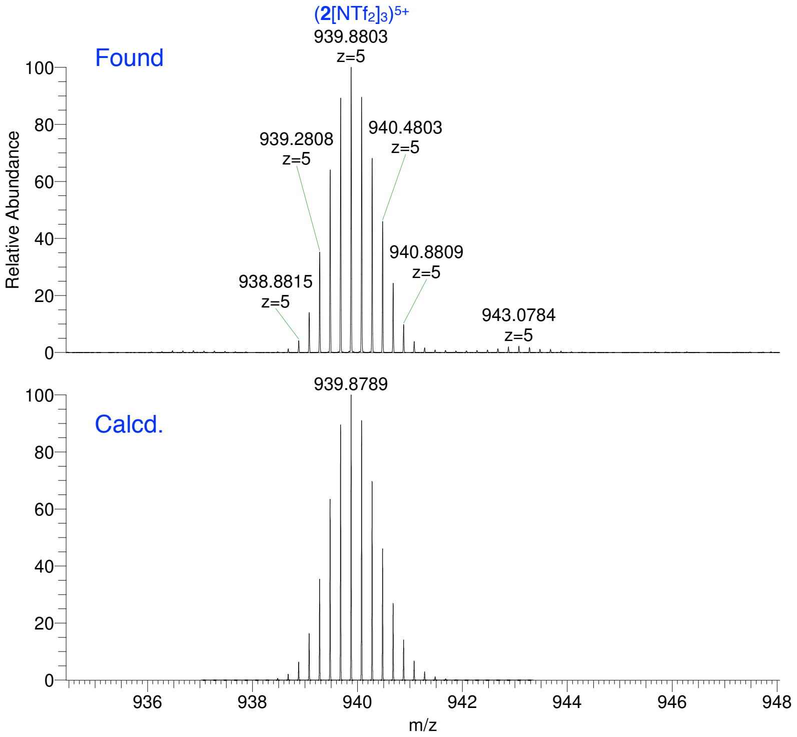

Figure S9 High resolution ESI-TOF-MS of **2**[NTf_2]$_8$ showing the +5 peak.

S3 X-ray crystallography of 2[NTf$_2$]$_8$

Crystals of **2**[NTf$_2$]$_8$·9.5C$_6$H$_6$·8.5CH$_3$CN·0.5H$_2$O were grown by vapour diffusion of benzene into an acetonitrile solution of **2**[NTf$_2$]$_8$. Data were collected at Beamline I19 of Diamond Light Source employing silicon double crystal monochromated synchrotron radiation (0.6889 Å) with ω and ψ scans at 100(2) K.[5] Data integration and reduction were undertaken with Xia2.[6-8] Subsequent computations were carried out using the WinGX-32 graphical user interface.[9] Multi-scan empirical absorption corrections were applied to the data using the AIMLESS[10] tool in the CCP4 suite.[11] The structure was solved by direct methods using SHELXT[12] then refined and extended with SHELXL.[13] In general, non-hydrogen atoms with occupancies greater than 0.5 were refined anisotropically. Carbon-bound hydrogen atoms were included in idealised positions and refined using a riding model. Disorder was modelled using standard crystallographic methods including constraints, restraints and rigid bodies where necessary. The asymmetric unit was found to contain half of a Fe$_4$L$_4$ assembly and associated counterions and solvent molecules. Bond lengths and angles within the two chemically identical organic ligands were restrained to be similar to each other and thermal parameter restraints (SIMU, RIGU) were applied to all atoms except for iron and sulfur.

The anions within the structure show evidence of substantial disorder. The four triflimide anions were modelled as disordered over five lattice sites including one site located on a special position with half occupancy. All the anions located on general positions were further modelled as disordered over two or three locations. The occupancies of the disordered anions were allowed to refine freely and then fixed at the obtained values. Some additional minor occupancy positions of the anions could not be located in the electron density map and were not included in the model resulting in a discrepancy of 0.975 counterions per asymmetric unit. Some lower occupancy disordered atoms were modelled with isotropic thermal parameters and bond length and thermal parameter restraints were applied to facilitate realistic modelling of the disordered triflimide anions. Bond length restraints were also applied to some solvent molecules and the benzene solvent molecules were modelled as rigid groups (AFIX 66).

CheckCIF gives two A and five B level alerts. These alerts (both A and B level) result from thermal motion and/or unresolved disorder of some anions and solvent molecules and the formula discrepancy as described above.

Crystallographic data have been deposited with the CCDC (CCDC 1881666).

Formula $C_{342}H_{311.50}B_4F_{48}Fe_4N_{40.50}O_{32.50}S_{16}$, M 7200.40, Monoclinic, space group

C 2/c (#15), a 35.953(7), b 38.642(8), c 27.135(5) Å, β 109.66(3), V 35500(14) Å^3, D_c 1.347 g cm^{-3}, Z 4, crystal size 0.012 by 0.010 by 0.010 mm, color purple, habit prism, temperature 100(2) Kelvin, λ(Synchrotron) 0.6889 Å, μ(Synchrotron) 0.318 mm^{-1}, T(Analytical)$_{min,max}$ 0.964377030078, 1.0, $2\theta_{max}$ 51.01, hkl range -44 44, -48 46, -33 33, N 179762, N_{ind} 36085(R_{merge} 0.0762), N_{obs} 19969(I > 2σ(I)), N_{var} 2633, residuals* $R1(F)$ 0.1096, $wR2(F^2)$ 0.3307, GoF(all) 1.057, $\Delta\rho_{min,max}$ -0.597, 0.878 e$^-$ Å^{-3}.

*$R1 = \Sigma||F_o| - |F_c||/\Sigma|F_o|$ for $F_o > 2\sigma(F_o)$; $wR2 = (\Sigma w(F_o^2 - F_c^2)^2/\Sigma(wF_c^2)^2)^{1/2}$ all reflections, $w=1/[\sigma^2(F_o^2)+(0.2000P)^2+20.0000P]$ where $P=(F_o^2+2F_c^2)/3$

S4 F⁻ binding of cage 2[NTf$_2$]$_8$

S4.1 NMR titration of 2[NTf$_2$]$_8$ with F⁻

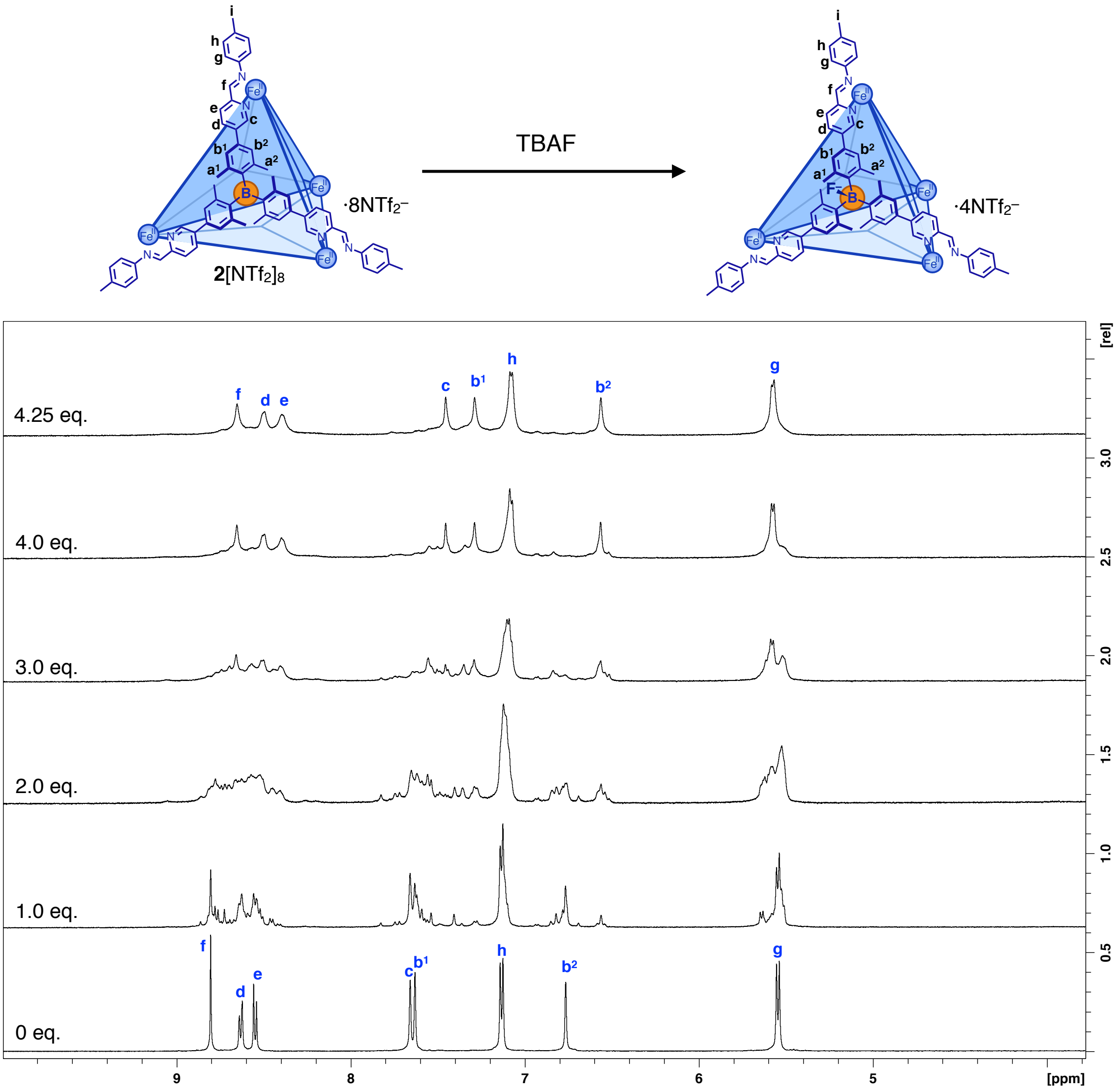

Figure S10 ^{1}H NMR (500 MHz) during the titration of **2**[NTf$_2$]$_8$ in CD$_3$CN with tetrabutylammonium fluoride (TBAF) trihydrate. The complexation of **2**[NTf$_2$]$_8$ with F⁻ was complete within 3 minutes after the addition of each equivalent of F⁻.

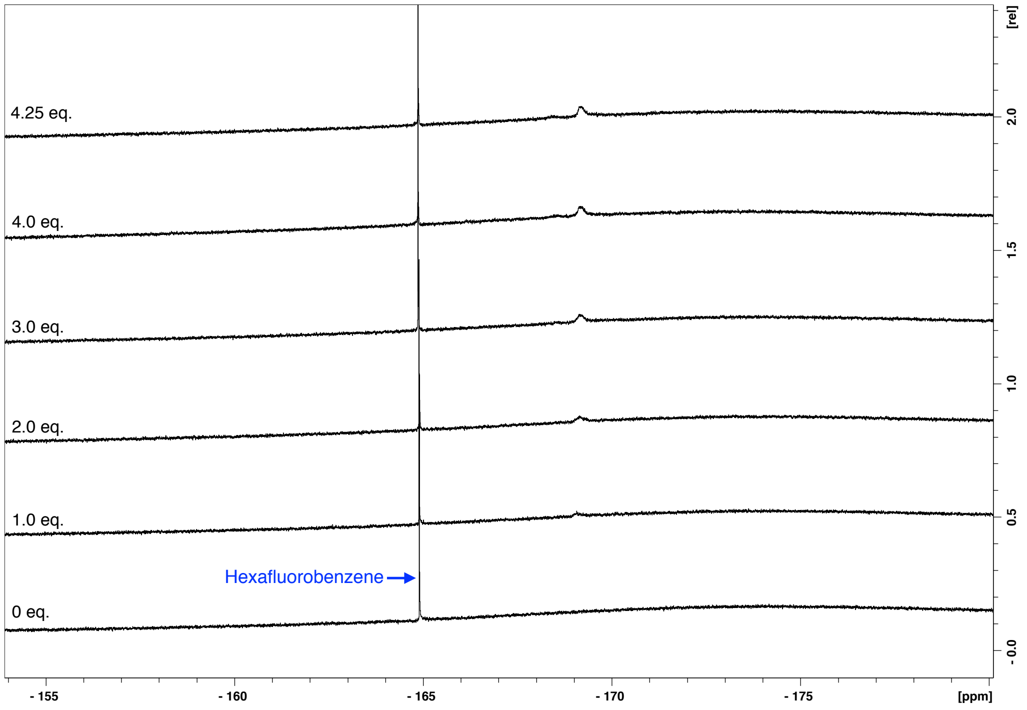

Figure S11 ^{19}F NMR (470 MHz) during the titration of **2**[NTf$_2$]$_8$ in CD$_3$CN with TBAF trihydrate.

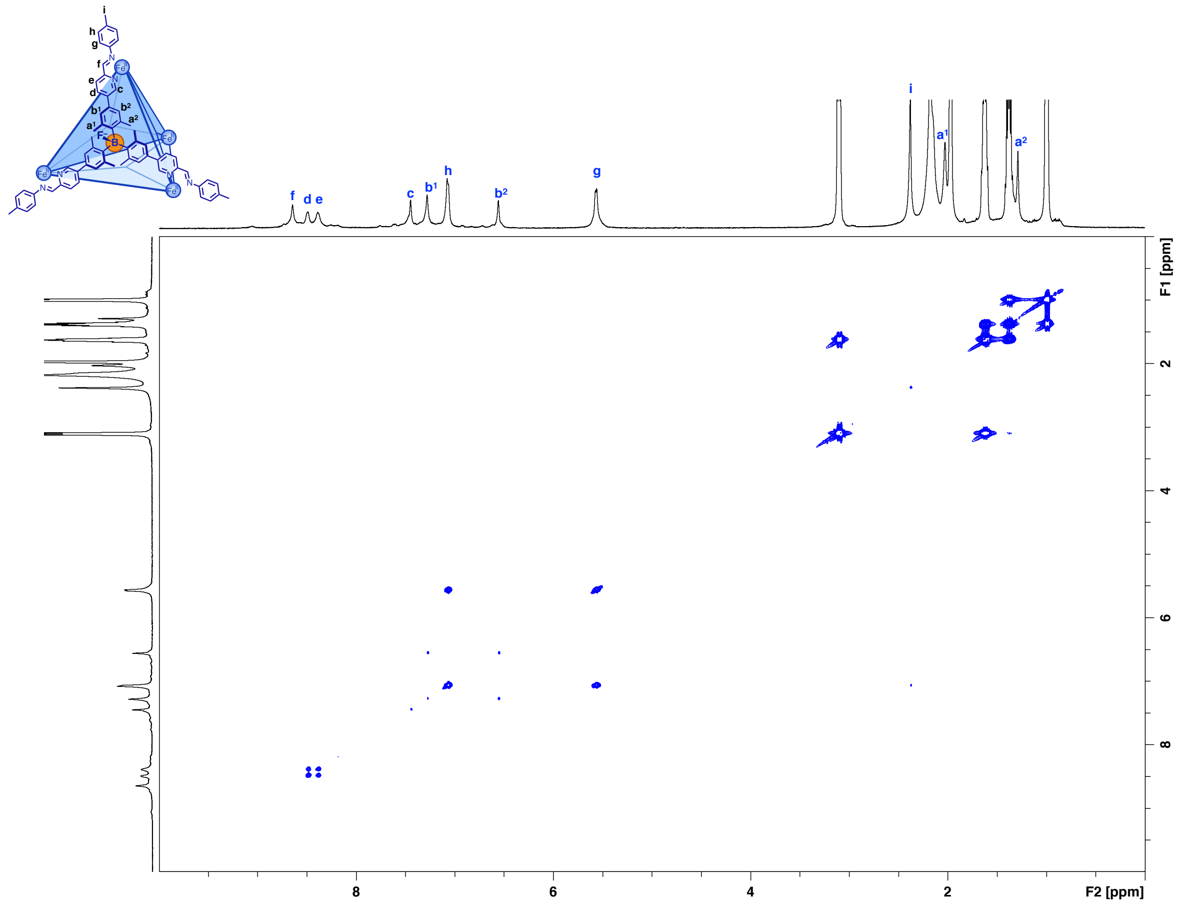

Figure S12 ^{1}H–^{1}H COSY (400 MHz) of **2**·F$_4$[NTf$_2$]$_4$ in CD$_3$CN at room temperature.

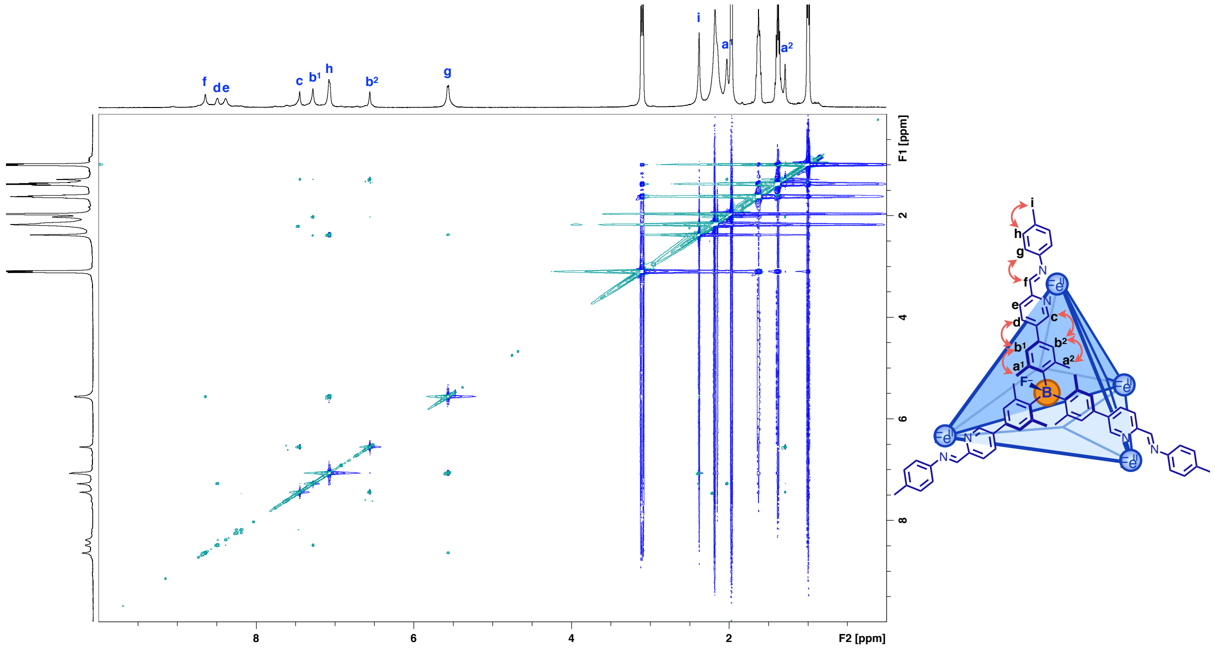

Figure S13 ^{1}H–^{1}H NOESY (400 MHz) of the mixture of **2**·$F_4[NTf_2]_4$ in CD_3CN at room temperature.

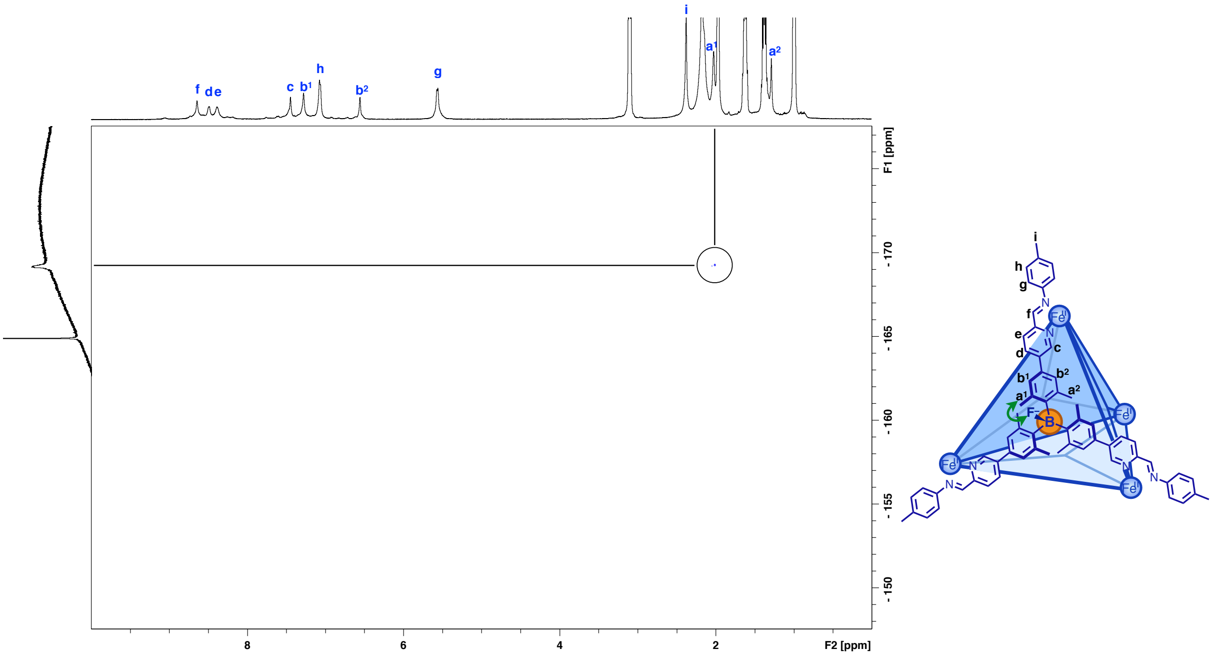

Figure S14 ^{1}H–^{19}F HOESY (400 MHz) of **2**·$F_4[NTf_2]_4$ in CD_3CN at room temperature.

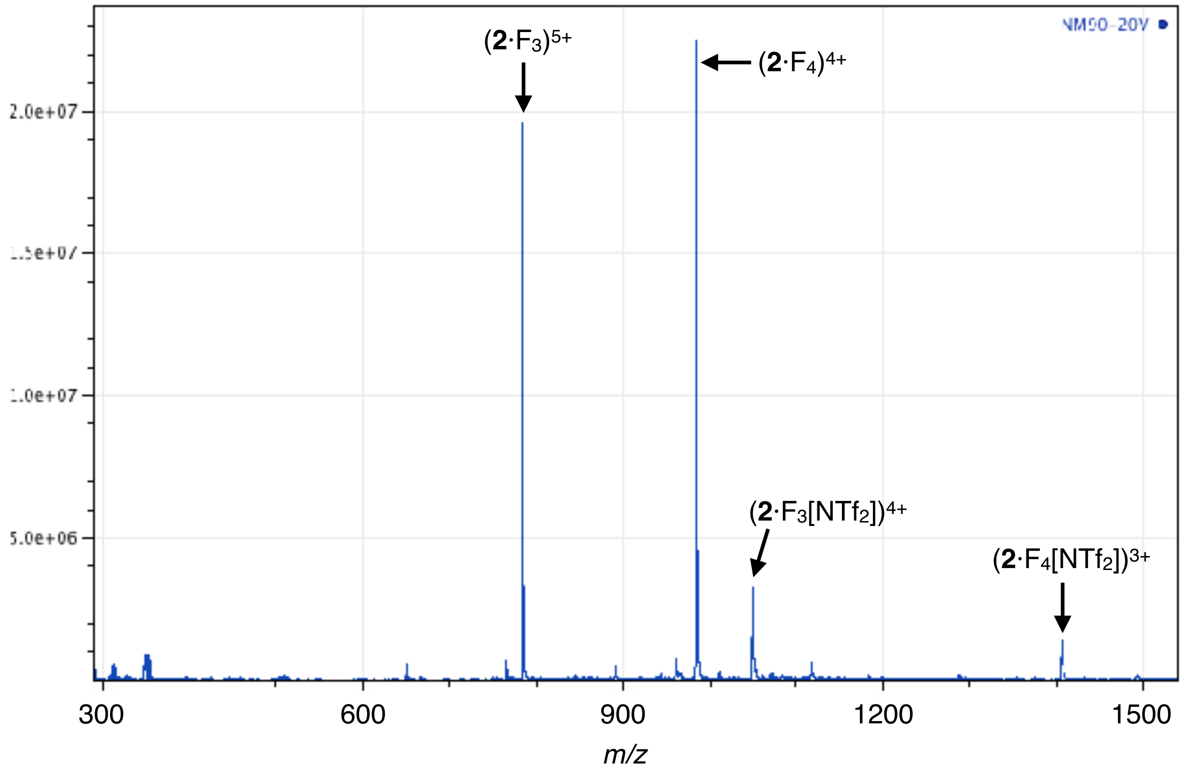

Figure S15 Low resolution ESI-TOF-MS of **2**·$F_4[NTf_2]_4$. LR-MS (ESI-TOF, positive) *m/z* calcd. for (**2**·$F_3)^{5+}$ 782.8, found 782.9; calcd. for (**2**·$F_4)^{4+}$ 983.3, found 983.4; calcd. for (**2**·$F_3[NTf_2])^{4+}$ 1048.6, found 1048.8; calcd. for (**2**·$F_4[NTf_2])^{3+}$ 1404.4, found 1404.5. The observation of **2**·F_3 is presumably due to fragmentation under the MS conditions.

S4.2 UV-Vis titration of 2[NTf$_2$]$_8$ with F$^-$

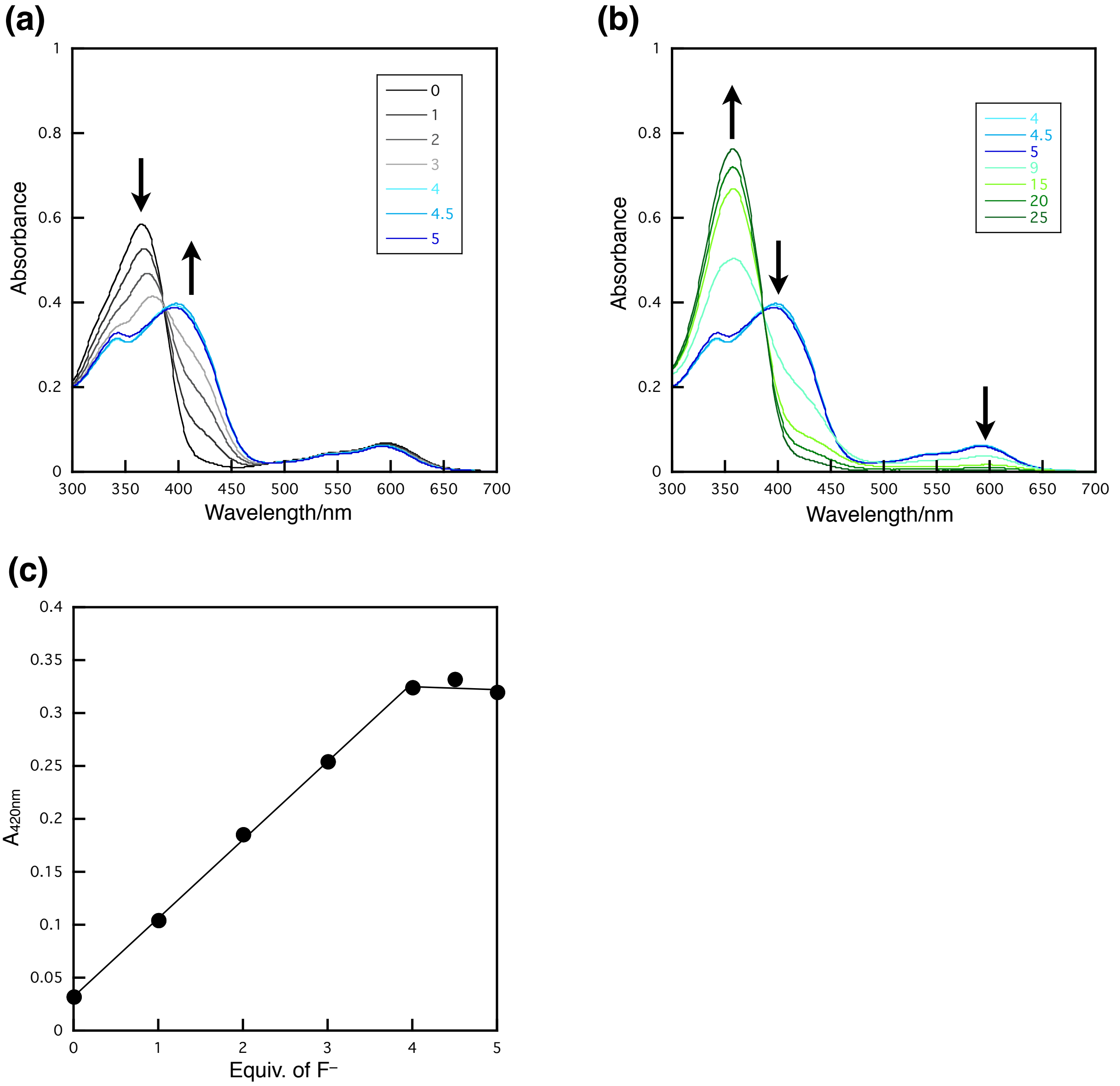

Figure S16 UV-Vis titration of **2**[NTf$_2$]$_8$ (20 μM in acetonitrile) with TBAF trihydrate. The spectral change during the addition of (a) 0 – 5 equiv and (b) 4 – 25 equiv of F$^-$. The loss of the MLCT band at around 500 – 600 nm was observed after the addition of excess F$^-$. We infer that coordination of F$^-$ to the Fe(II) ions leads to gradual decomposition of **2** and generation of the free ligand. (c) Plot of absorbance at 420 nm vs equivalents of F$^-$ (0 – 5 equiv).

S5 Removal of F⁻ from 2·$F_4[NTf_2]_4$

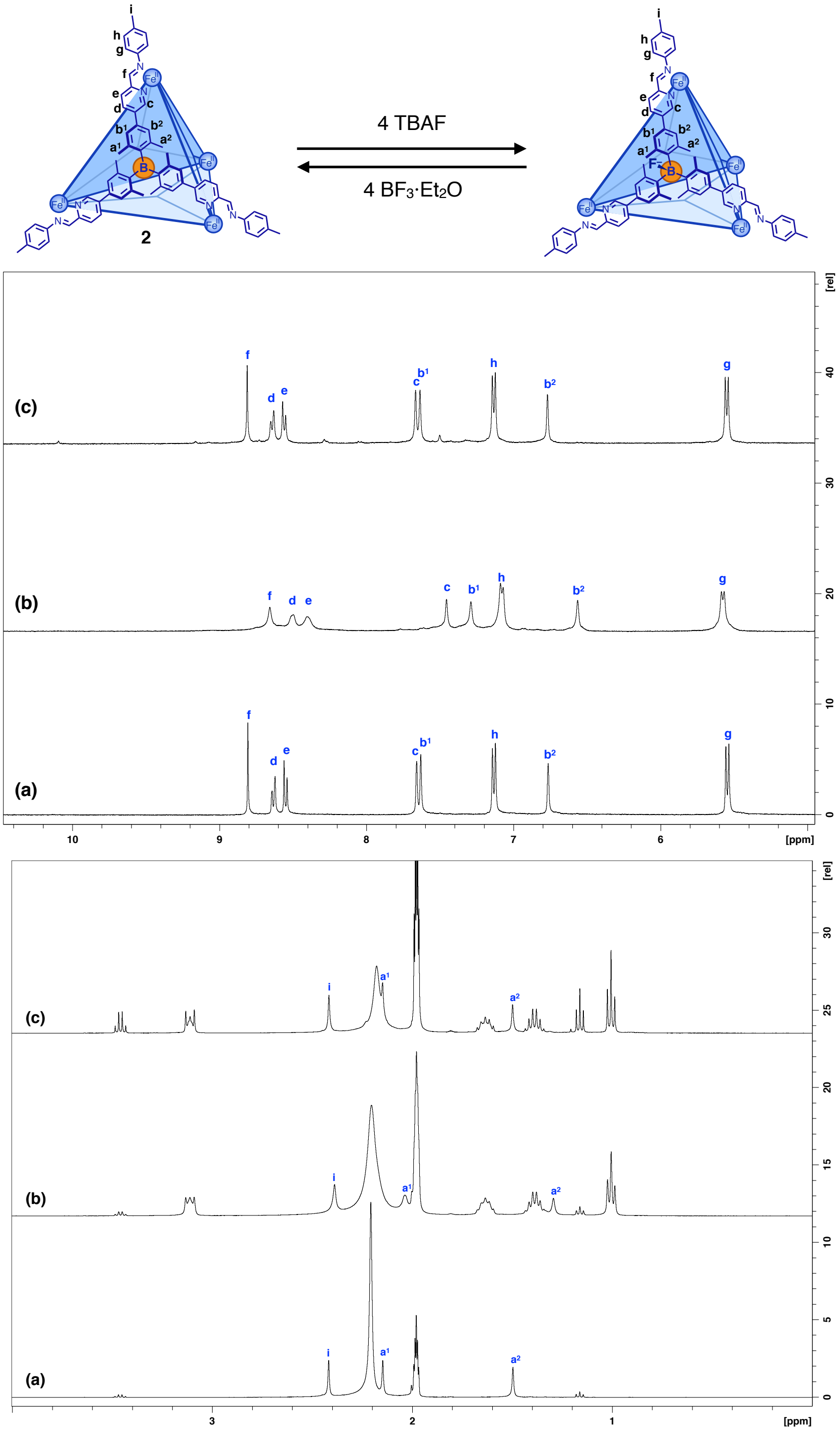

Figure S17 Removal of F^- from **2**·$F_4[NTf_2]_4$ by $BF_3·Et_2O$ in CD_3CN at room temperature. ^1H NMR spectrum (400 MHz) of (a) **2**$[NTf_2]_8$, (b) **2**·$F_4[NTf_2]_4$, and (c) the reaction mixture of **2**·$F_4[NTf_2]_4$ with $BF_3·Et_2O$ (4.0 equiv).

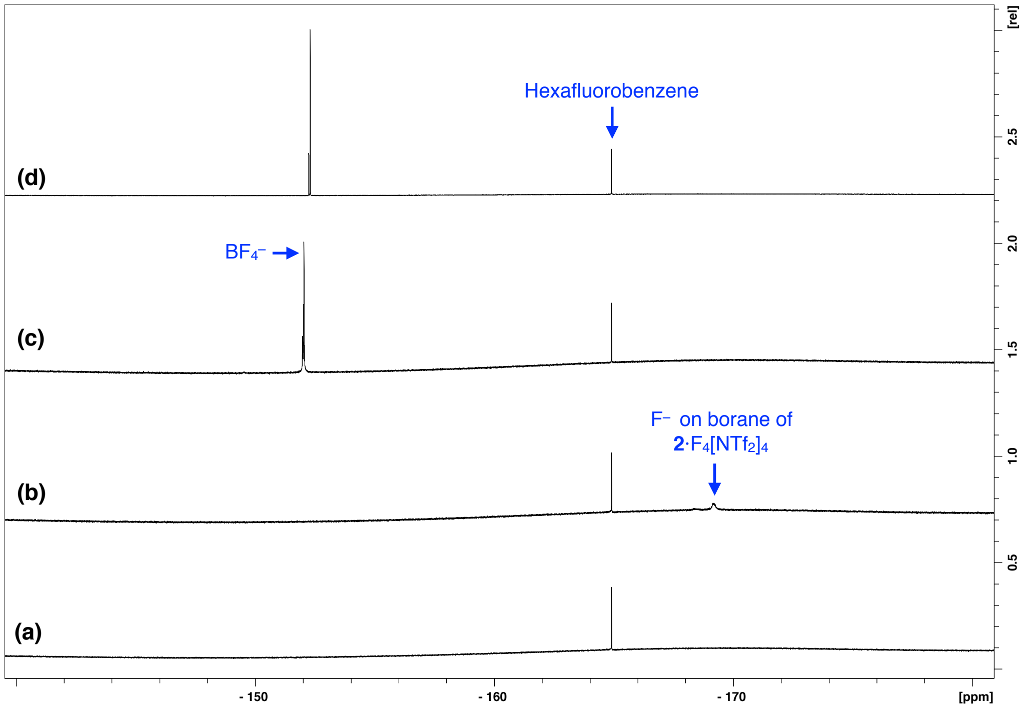

Figure S18 Removal of F⁻ from **2**·$F_4[NTf_2]_4$ by $BF_3·Et_2O$ in CD_3CN at room temperature. ^{19}F NMR spectrum (376 MHz) of (a) **2**$[NTf_2]_8$, (b) **2**·$F_4[NTf_2]_4$, (c) the reaction mixture of **2**·$F_4[NTf_2]_4$ with $BF_3·Et_2O$ (4.0 equiv), and (d) tetrabutylammonium tetrafluoroborate.

S6 Counter anion exchange of $2[NTf_2]_8$ to $2[BAr_{f6}]_8$

To a solution of **2**$[NTf_2]_8$ (10 mg, 1.6 μmol) and $NaBAr_{f6}$ (43 mg, 15 μmol) in acetone (1.5 mL), water (0.5 mL) was added. The resulting precipitate was collected by centrifugation and washed by acetone (1.0 mL × 2) to obtain **2**$[BAr_{f6}]_8$ (40 mg, 92%). ^1H NMR (*δ* (ppm), 400 MHz, perfluoromethylcyclohexane (reference: CD_3CN capillary), 338K: 8.14 – 7.83 (br, 12H), 7.57 – 6.91 (m, 100H), 6.91 – 6.67 (br, 32H), 6.67 – 6.47 (br, 12H), 6.47 – 6.19 (br, 24H), 6.19 – 5.84 (br, 12H), 4.95 – 4.52 (br, 24H), 1.78 – 1.44 (br, 36H), 1.37 – 1.18 (br, 36H), 0.86 – 0.55 (br, 36H).

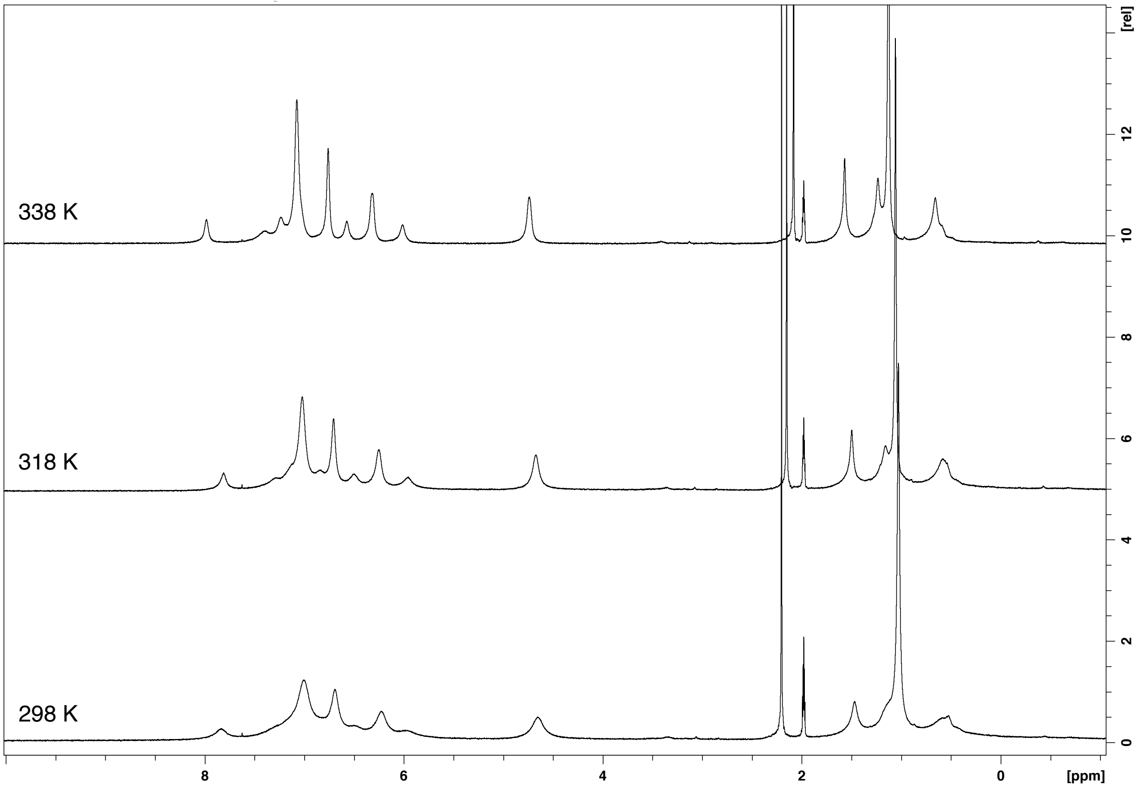

Figure S19 VT ^1H NMR (400 MHz) of **2**$[BAr_{f6}]_8$ in perfluoromethylcyclohexane. Broadening of the spectrum was observed at room temperature but the spectrum became sharper when the temperature was increased. We infer that the broadening of the spectrum is caused by aggregation of the cage at lower temperatures.

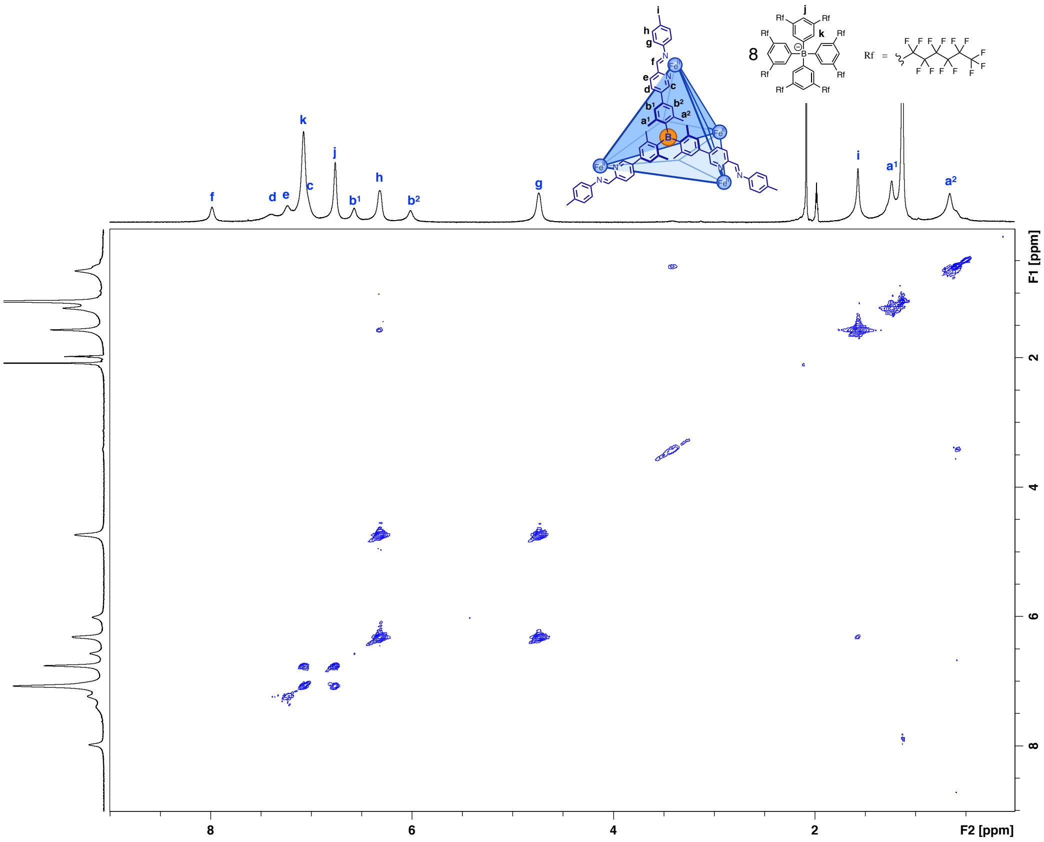

Figure S20 ^1H–^1H COSY (400 MHz) of **2**[BAr$_{f6}$]$_8$ in perfluoromethylcyclohexane at 338 K.

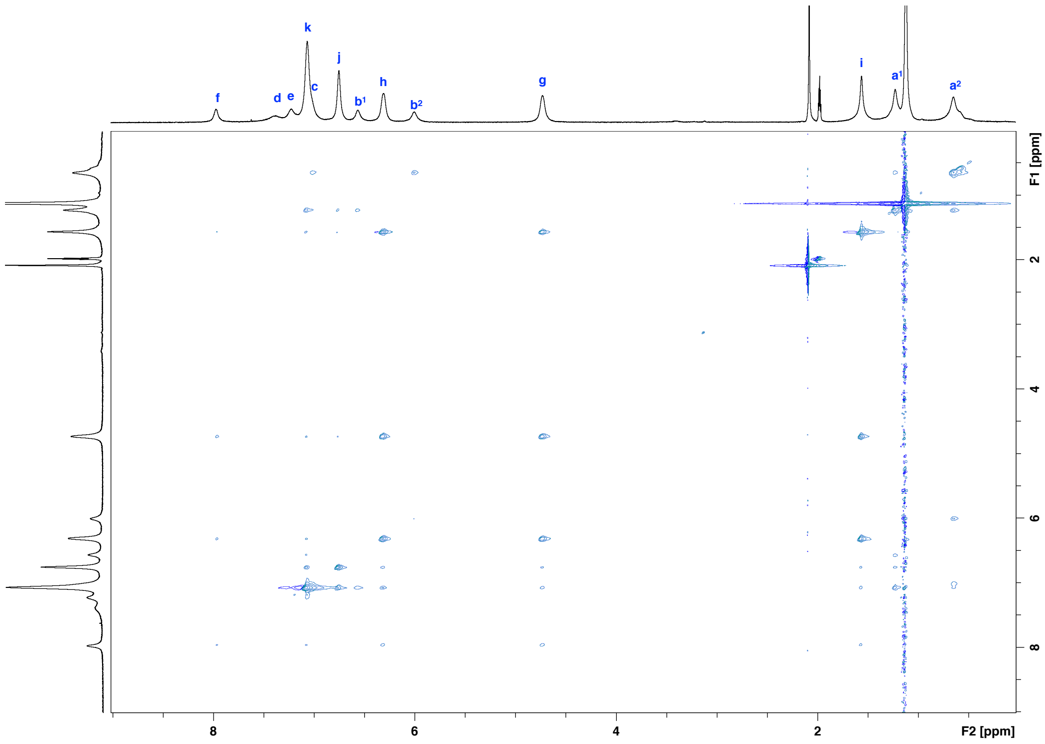

Figure S21 ^1H–^1H NOESY (400 MHz) of **2**[BAr$_{f6}$]$_8$ in perfluoromethylcyclohexane at 338 K.

S7 Transport experiment of 2 within a triphasic solvent system

S7.1 Calibration curves for transport experiment

The calibration curves of **2**[BAr$_{f6}$]$_8$ in perfluoromethylcyclohexane and **2**[SO$_4$]$_4$ in water containing 25% acetonitrile were obtained to determine the relationship between the concentration and the UV-Vis absorbance of **2** for the transport experiment. A stock solution of **2**[BAr$_{f6}$]$_8$ in perfluoromethylcyclohexane (125 μM) was prepared and diluted appropriately, and the UV-Vis spectrum of each solution (10 – 50 μM) was recorded (Fig. S22(a)). A stock solution of **2**[SO$_4$]$_4$ in water containing 25% acetonitrile was prepared by the following procedure. To a biphasic system composed of **2**[BAr$_{f6}$]$_8$ in perfluoromethylcyclohexane (125 μM, 1.0 mL) and water containing 25% acetonitrile (1.0 mL), tetrabutylammonium sulfate (TBA$_2$SO$_4$) (4.0 equiv with respect to **2**) was added to transfer **2** into water containing 25% acetonitrile. The stock solution was diluted appropriately, and the UV-Vis spectrum of each solution (10 – 50 μM) was recorded (Fig. S22(b)).

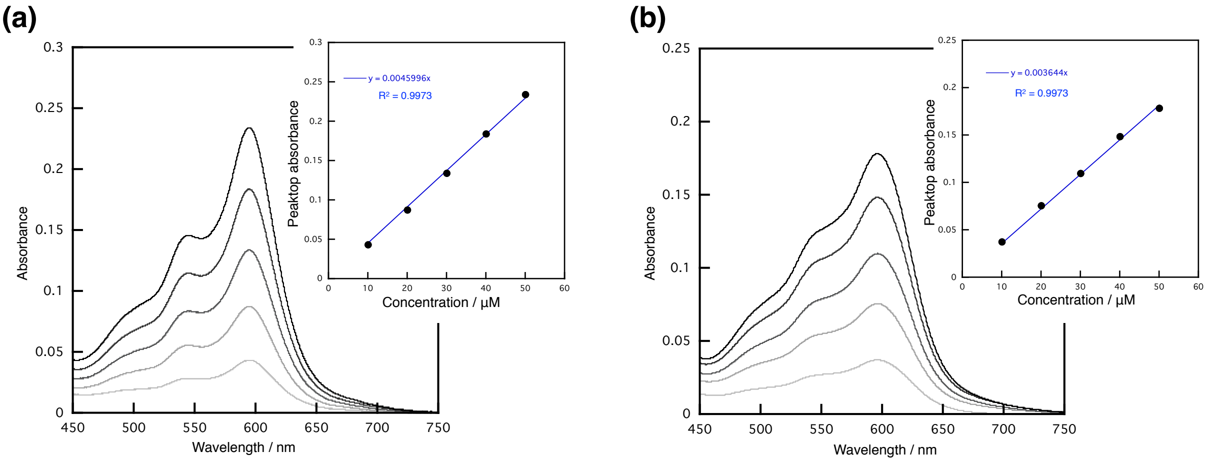

Figure S22 UV-Vis data and calibration curve of (a) **2**[BAr$_{f6}$]$_8$ in perfluoromethylcyclohexane and (b) **2**[SO$_4$]$_4$ in water containing 25% acetonitrile.

S7.2 UV-Vis titration of 2 with TBAF for transport experiment

To estimate the number of bound F^- on **2** in the DCP phase during the phase transfer process, UV-Vis titration of **2**[NTf_2]$_8$ against TBAF was performed in 2,2-dichloropropane/acetonitrile = 5 : 1 (*v*/*v*) (Fig. S23(a)). To a solution of **2**[NTf_2]$_8$ (40 μM), 0 – 4.5 equiv of TBAF (6.0 mM) was added. The absorption maximum wavelength of the MLCT of each spectrum (around 600 nm) was monitored to determine the relationship with the amount of added F^-. As a result, a linear relationship between added F^- and absorption maximum wavelength was observed in the range of 0 – 4.0 equiv of F^- (Fig. S23(b)). Based on this linear relationship, the average number of F^- on **2** during the transport experiment was roughly estimated as shown in Fig. S24(c) and Fig. S25(c).

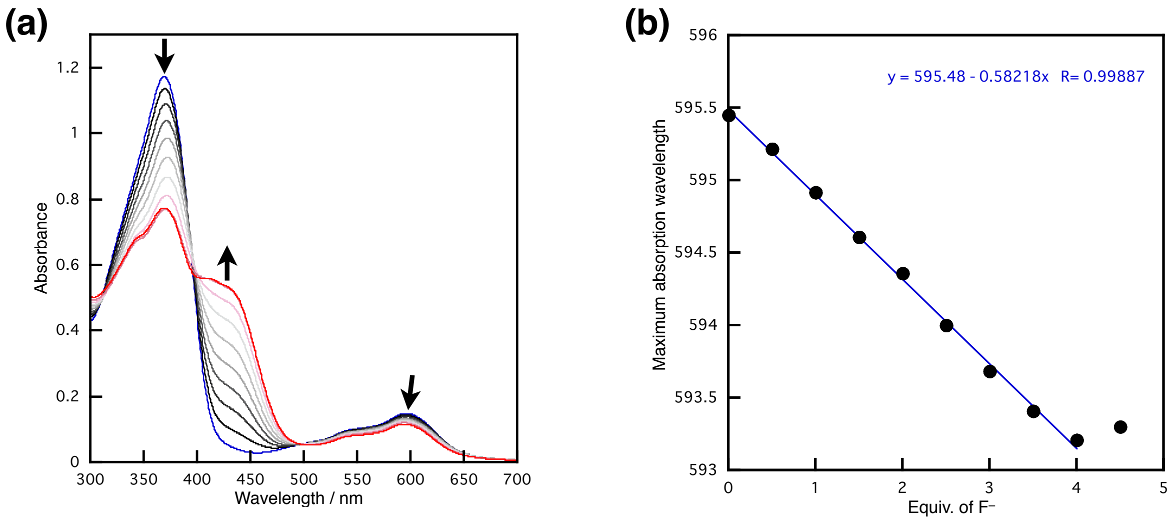

Figure S23 The UV-Vis titration of **2**[NTf_2]$_8$ (40 μM) with TBAF trihydrate (0 – 4.5 equiv with respect to **2**) in 2,2-dichloropropane/acetonitrile = 5 : 1 (*v*/*v*).

S7.3 Transport experiment of 2 in a microtube

S7.3.1 Experimental procedure

The following stock solutions of ions were prepared; TBAF trihydrate in dichloromethane (100 mM), TBA_2SO_4 in water containing 25% acetonitrile (125 mM), magnesium sulfate in water containing 25% acetonitrile (50.0 mM), $NaBAr_{f6}$ in perfluoromethylcyclohexane (29.6 mM). The triphasic system composed of perfluoromethylcyclohexane solution containing **2**$[BAr_{f6}]_8$ (PFMC, 125 μM, 200 μL), 2,2-dichloropropane (DCP, 200 μL), and water containing 25% acetonitrile (Aq, 200 μL) were prepared in a microtube. After the addition of each ion, the tube was shaken for 1 – 2 min and centrifuged for 1 min. The minimum proportion of ions required to cause phase transfer was added in each case.

Each stock solution was added to the microtube in the following order;

Direction 1 (PFMC → DCP → Aq → PFMC → DCP → Aq, Fig. S24); TBAF 4.0 equiv → TBA_2SO_4 3.0 equiv and $MgSO_4$ 2.0 equiv → $NaBAr_{f6}$ 16.0 equiv → TBAF 8.0 equiv → TBA_2SO_4 12.5 equiv and $MgSO_4$ 4.0 equiv.

Direction 2 (PFMC → Aq → DCP → PFMC → Aq → DCP, Fig. S25); TBA_2SO_4 4.0 equiv → TBAF 8.0 equiv and $NaBAr_{f6}$ 8.9 equiv → $NaBAr_{f6}$ 7.7 equiv and $MgSO_4$ 4.0 equiv → TBA_2SO_4 6.3 equiv → TBAF 8.0 equiv and $NaBAr_{f6}$ 8.9 equiv.

To record the UV-Vis spectra, a sample of each step ((i) – (vi)) was prepared separately and each phase was diluted so that the concentration of **2** in the ideal case where all of **2** used in this experiment exists in the phase would be 40 μM. The PFMC and the aqueous phases were diluted with perfluoromethylcyclohexane and water containing 25% acetonitrile respectively. The DCP phase were diluted with 2,2-dichloropropane and acetonitrile so that the solvent of the UV-Vis sample would be 2,2-dichloropropane/acetonitrile = 5 : 1 (*v*/*v*).

S7.3.2 Experimental results and discussion about transfer process

S7.3.2.1 The structure and the counter anions of 2 in each phase

According to the comparison of UV-Vis spectra of each phase (Fig. S24(b) – (d), S25(b) – (d)) and the UV-Vis titration result with F^- (Fig. S23), it was revealed that **2** has bound F^- in DCP but does not bind F^- in PFMC and in the aqueous phase. The average number of F^- on **2** in DCP was roughly estimated to be 2.6 – 3.8 equiv (Fig. S24(c), Fig. S25(c)) as deduced from the absorption maximum wavelength of MLCT of each spectrum as described in Section S7.2. This incomplete binding of F^- is presumably because of the competition between F^- binding by **2** and stabilization of F^- by water in the aqueous phase, since F^- is highly hydrophilic.

We infer that when **2** has more BAr_{f6}^- as the counter anion, it can be solubilized in PFMC because of the high content ratio of fluorine within the whole complex. In contrast, when some of the BAr_{f6}^- are replaced by F^-, the ratio of fluorine is no longer sufficient to solubilize the cage in PFMC, so that it is more stabilized in DCP compared to PFMC. In DCP when both BAr_{f6}^- and SO_4^{2-} are present, we infer that **2** has BAr_{f6}^- as the counter anion, since SO_4^{2-} has low affinity for non-polar organic solvents such as halogenated solvents. This was further confirmed by the ESI-MS of DCP, where F^- adducts of **2** were observed with BAr_{f6}^- but not with SO_4^{2-} as the counter anion (Fig. S26).

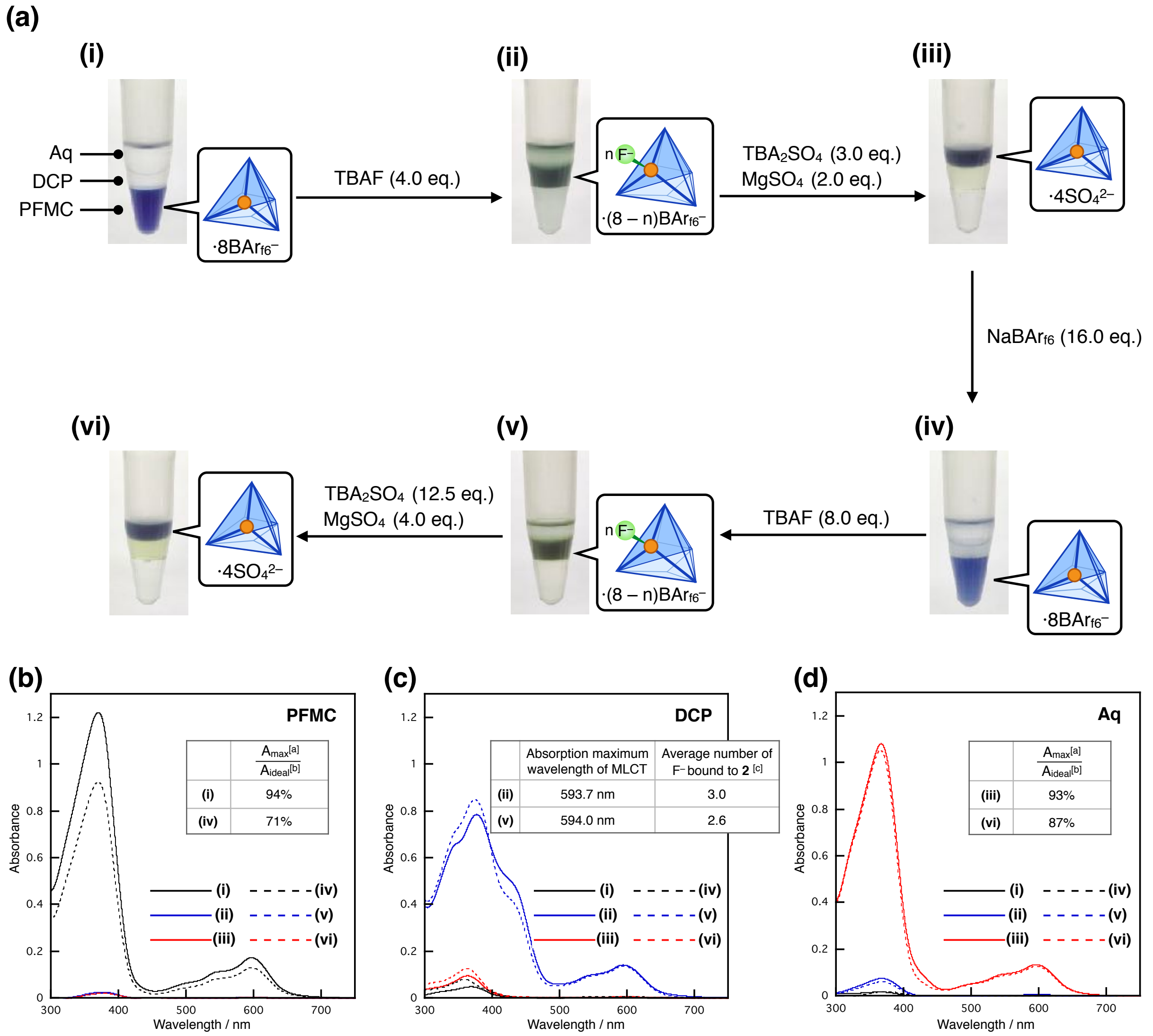

Figure S24 The transport experiment of **2** (direction 1). (a) Photos of each step ((i) – (vi)) of the experiment. UV-Vis spectra of (b) PFMC, (c) DCP, and (d) the aqueous phase of each step ((i) – (vi)) of the experiment. The data of A_{max} / A_{ideal} and estimated average number of F^- on **2** are also included in each figure.

[a] A_{max} is the absorption maximum of MLCT (around 600 nm) of each spectrum of the transport experiment.

[b] A_{ideal} is the absorption maximum of the MLCT in the ideal case where all of **2** used in this experiment exists in the phase, which was obtained by using the calibration curves shown in Figure S22. Thus, A_{max} / A_{ideal} represents the percentage of **2** that exists in each phase. A_{ideal} of the DCP phase could not be obtained since it changes according to the number of bound F^- on **2**.

[c] The average number of F^- bound to **2** was estimated according to the procedure described in Section S7.2.

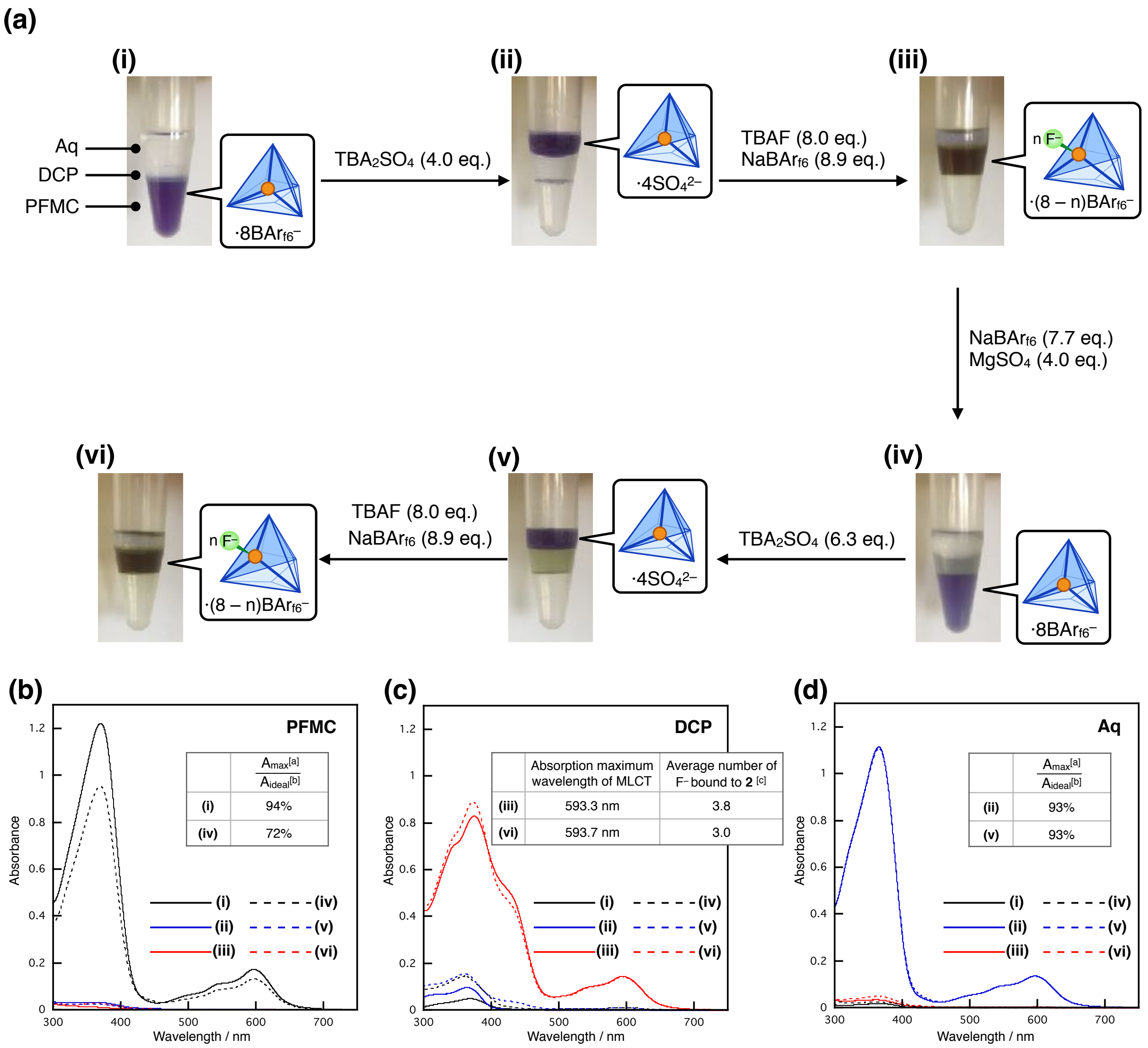

Figure S25 The transport experiment of **2** (direction 2). (a) Photos of each step ((i) – (vi)) of the experiment. UV-Vis spectra of (b) PFMC, (c) DCP, and (d) the aqueous phase of each step ((i) – (vi)) of the experiment. The data of A_{max} / A_{ideal} and estimated average number of F^- on **2** are included in each figure.

[a] A_{max} is the absorption maximum of MLCT (around 600 nm) of each spectrum of the transport experiment.

[b] A_{ideal} is the absorption maximum of the MLCT in the ideal case where all of **2** used in this experiment exists in the phase, which was obtained by using the calibration curves shown in Figure S22. Thus, A_{max} / A_{ideal} represents the percentage of **2** that exists in each phase. A_{ideal} of the DCP phase could not be obtained since it changes according to the number of bound F^- on **2**.

[c] The average number of F^- bound to **2** was estimated according to the procedure described in Section S7.2.

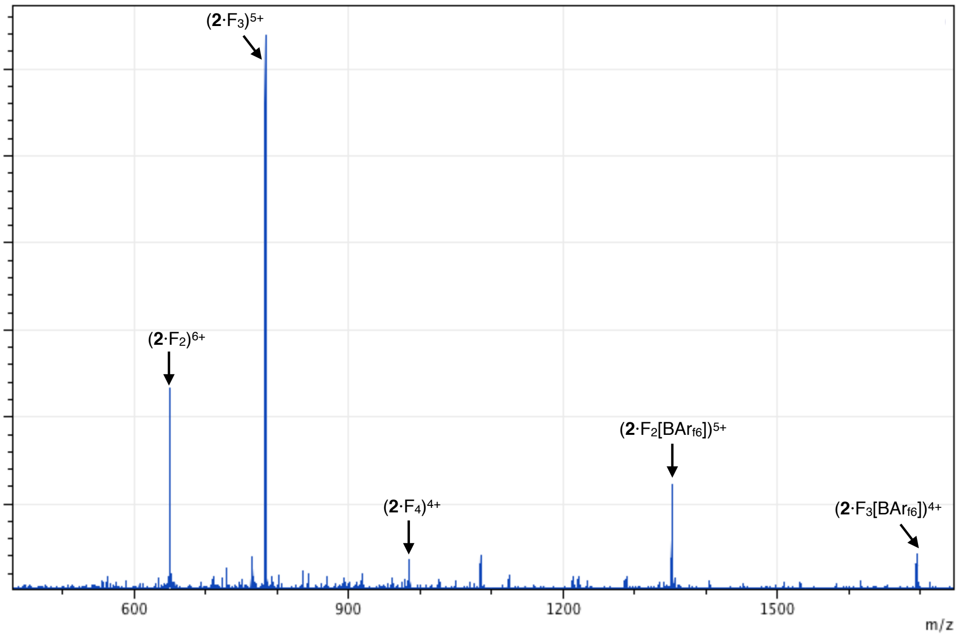

Figure S26 Low resolution ESI-MS of DCP phase of state (iii) in Figure S25(a). LR-MS (ESI-TOF, positive) *m/z* calcd. for $(\mathbf{2}\cdot F_2)^{6+}$ 649.2, found 649.3; calcd. for $(\mathbf{2}\cdot F_3)^{5+}$ 782.8, found 783.1; calcd. for $(\mathbf{2}\cdot F_4)^{4+}$ 983.3, found 983.7; calcd. for $(\mathbf{2}\cdot F_2[BAr_{f6}])^{5+}$ 1351.7, found 1352.0; calcd. for $(\mathbf{2}\cdot F_3[BAr_{f6}])^{4+}$ 1694.4, found 1694.7.

S7.3.2.2 F^- removal process

Mg^{2+} was added to remove F^- from the system to avoid the gradual decomposition of **2** provoked by excess F^-. We propose that the process of this F^- removal occurs as shown in Figure S27. In the case of the transport experiment shown in Figure S24 (direction 1, (ii) → (iii) and (v) → (vi)), the addition of SO_4^{2-} causes the transfer of **2**·$F_n[BAr_{f6}]_{8-n}$ from DCP to the aqueous phase through the formation of **2**$[SO_4]_4$. F^- is dissociated from **2** because of weaker B-F^- interactions in aqueous solution[14] and then precipitates as MgF_2. We infer that the direct removal of F^- from **2**·$F_n[BAr_{f6}]_{8-n}$ in DCP by Mg^{2+} in the aqueous phase is slower because of the poor solubility in the DCP phase where the cage resides. Similarly in the case of the experiment shown in Figure S25 (direction 2, (iii) → (iv)), the addition of BAr_{f6}^- causes the transfer of **2**·$F_n[BAr_{f6}]_{8-n}$ from DCP to PFMC through the formation of **2**$[BAr_{f6}]_8$ and dissociation of F^- which precipitates as MgF_2.

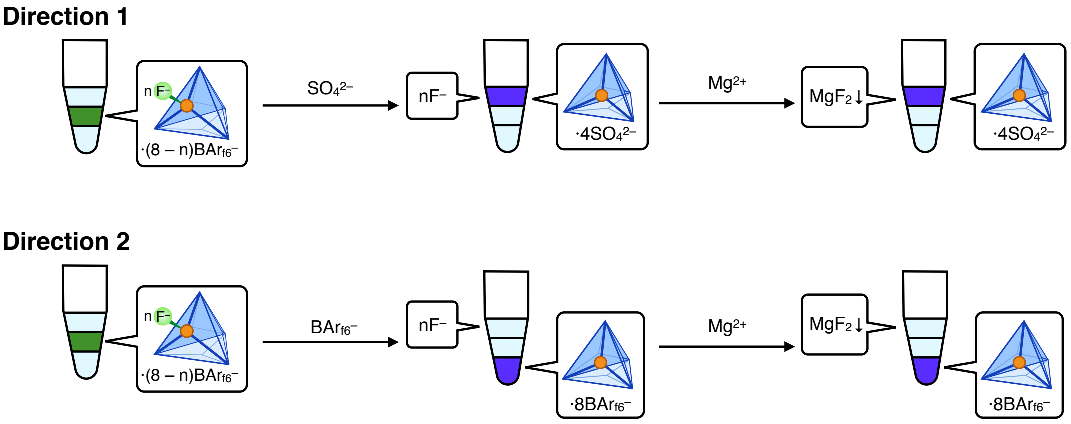

Figure S27 The proposed process of F^- removal during the transport experiment.

S7.3.2.3 The efficiency of each transfer step

The value of A_{max} / A_{ideal} (Figure S24 and S25), which represents the percentage of **2** existing in the targeted phases, remained high after transport in both directions. Although some emulsions involving insoluble material appeared at the point of (iv) in both directions, which causes a decrease in the UV-Vis intensity ((i) → (iv) : 94% to 71% in Fig. S24(b), (i) → (iv) : 94% to 72% in Fig. S25(b)), they were observed to redissolve after the subsequent addition of TBAF or TBA_2SO_4 as confirmed by the recovery of the UV-Vis intensity ((vi) : 87% in Fig. S24(d), (v) : 93% in Fig. S25(d)). Considering that the decomposition of the cage structure, which leads to the generation of the free ligand, causes an irreversible decrease in MLCT intensity, we infer that the majority of **2** retained their structure during the transfer process. We assume that the temporary insoluble material at the point of (iv) could be caused by incomplete anion exchange and might be decreased by changing the solvent combinations to further improve the transfer efficiency.

S7.3.2.4 The rate of anion exchange and phase transfer

In the case of transfer experiments in the microtubes, each phase transfer step occurred within 1 – 2 minutes of agitation. Although the rapid nature of these processes precluded further detailed analysis of the kinetics, this result corresponds well with our previous reports showing the fast exchange of non-coordinating counter anions of cages.[15] We also confirmed that the complexation of cage **2** with F^- occurred within 3 minutes in the separate NMR titration experiment shown in Figures S10 and S11. In the circular glass tube, the phase transfer step took approximately 3 minutes of agitation. We infer that the mixing of phases in the circular tube is less efficient compared to the microtube, due to the smaller diameter of the circular glass tube.

S7.4 Transport experiment in a circular glass tube

The circular glass tube designed for this experiment had an internal diameter of 4 mm. This glass tube has three injection points, each of which can be closed by the rubber septa so that the solution of chemical signals can be added by syringe to either of the phases.

The same stock solutions of ions as the experiment in the microtube were used for this experiment. The triphasic system composed of perfluoromethylcyclohexane solution containing 125 μM of **2**$[BAr_{f6}]_8$ (PFMC, 400 μL), 2,2-dichloropropane (DCP, 400 μL), and water containing 25% acetonitrile (Aq, 550 μL) was prepared. After the addition of each salt solution, the tube was shaken for 3 min to allow the phases to mix. The stock solutions of each ion were directly added to the targeted phases from either of the three injection points of the glass tube. Each stock solution was added to the glass tube in the following order; Direction 1 (PFMC → DCP → Aq, Fig. 5a-b); TBAF 4.0 equiv → TBA_2SO_4 3.0 equiv and $MgSO_4$ 2.0 equiv. Direction 2 (PFMC → Aq → DCP, Fig. 5c-d); TBA_2SO_4 4.0 equiv → TBAF 8.0 equiv and $NaBAr_{f6}$ 8.9 equiv.

S8 References

(1) Li, J.; Zhang, G.; Zhang, D.; Zheng, R.; Shi, Q.; Zhu, D. Boron-containing monopyrrolo-annelated tetra thiafulvalene compounds: synthesis and absorption spectral/electrochemical responsiveness toward fluoride ion. *J. Org. Chem.* **2010**, *75*, 5330-5333.

(2) Sibi, M. P.; Petrovic, G. Enantioselective radical reactions: the use of metal triflimides as Lewis acids. *Tetrahedron: Asymmetry* **2003**, *14*, 2879–2882.

(3) Van Den Broeke, J.; Deelman, B.-J.; van Koten, G. Tetrakis{3,5-bis(perfluorohexyl)phenyl}borate: a highly fluorous anion. *Tetrahedron Lett.* **2001**, *42*, 8085-8087.

(4) Plajer, A. J.; Percástegui, E. G.; Santella, M.; Rizutto, F. J.; Gan, Q.; Laursen, B. W.; Nitschke, J. R. Fluorometric recognition of nucleotides within a water-soluble tetrahedral capsule. *Angew. Chem. Int. Ed.* **2019**, *58*, 4200-4204.

(5) Allan, D.; Nowell, H.; Barnett, S.; Warren, M.; Wilcox, A.; Christensen, J.; Saunders, L.; Peach, A.; Hooper, M.; Zaja, L.; Patel, S.; Cahill, L.; Marshall, R.; Trimnell, S.; Foster, A.; Bates, T.; Lay, S.; Williams, M.; Hathaway, P.; Winter, G.; Gerstel, M.; Wooley, R. A novel dual air-bearing fixed-χ diffractometer for small-molecule single-crystal X-ray diffraction on beamline I19 at Diamond Light Source. *Crystals* **2017**, *7*, 336.

(6) Collaborative Computational Project, N. The CCP4 suite: programs for protein crystallography. *Acta Cryst.* **1994**, *D50*, 760-763.

(7) Evans, P. Scaling and assessment of data quality. *Acta Cryst.* **2006**, *D62*, 72-82.

(8) Winter, G. xia2: an expert system for macromolecular crystallography data reduction. *J. Appl. Crystallogr.* **2010**, *43*, 186-190.

(9) Farrugia, L. WinGX and ORTEP for Windows: an update. *J. Appl. Crystallogr.* **2012**, *45*, 849-854.

(10) Evans, P. R.; Murshudov, G. N. How good are my data and what is the resolution? *Acta Cryst.* **2013**, *D69*, 1204-1214.

(11) Winn, M. D.; Ballard, C. C.; Cowtan, K. D.; Dodson, E. J.; Emsley, P.; Evans, P. R.; Keegan, R. M.; Krissinel, E. B.; Leslie, A. G. W.; McCoy, A.; McNicholas, S. J.;

Murshudov, G. N.; Pannu, N. S.; Potterton, E. A.; Powell, H. R.; Read, R. J.; Vagin, A.; Wilson, K. S. Overview of the CCP4 suite and current developments. *Acta Cryst.,* **2011**, *D67*, 235-242.

(12) Sheldrick, G. M. SHELXT - Integrated space-group and crystal-structure determination. *Acta. Cryst.* **2015**, *A71*, 3-8.

(13) Sheldrick, G. M. Crystal structure refinement with SHELXL. *Acta. Cryst.* **2015**, *C71*, 3-8.

(14) Huh, J. O.; Kim, H.; Lee, K. M.; Lee, Y. S.; Do, Y.; Lee, M. H. *o*-Carborane-assisted Lewis acidity enhancement of triarylboranes. *Chem. Commun.*, **2010**, *46*, 1138-1140.

(15) (a) Rizzuto, F. J.; Wu, W-Y.; Ronson, T. K.; Nitschke, J. R. Peripheral templation generates an $M^{II}_6L_4$ guest-binding capsule. *Angew. Chem. Int. Ed.*, **2016**, *55*, 7958-7962. (b) Percástegui, E. G.; Mosquera, J.; Ronson, T. K.; Plajer, A. J.; Kieffer, M.; Nitschke, J. R. Waterproof architectures through subcomponent self-assembly. *Chem. Sci.*, **2019**, *10*, 2006-2018.
